# Relative abundance of ‘*Candidatus* Tenderia electrophaga’ is linked to cathodic current in an aerobic biocathode community

**DOI:** 10.1111/1751-7915.12757

**Published:** 2017-07-11

**Authors:** Anthony P. Malanoski, Baochuan Lin, Brian J. Eddie, Zheng Wang, W. Judson Hervey, Sarah M. Glaven

**Affiliations:** ^1^ United States Naval Research Laboratory 4555 Overlook Ave. SW Washington DC 20375 USA; ^2^ Defense Threat Reduction Agency 8725 John J Kingman Rd #6201 Fort Belvoir VA 22060 USA

## Abstract

Biocathode microbial communities are proposed to catalyse a range of useful reactions. Unlike bioanodes, model biocathode organisms have not yet been successfully cultivated in isolation highlighting the need for culture‐independent approaches to characterization. Biocathode MCL (*Marinobacter*,* Chromatiaceae*,* Labrenzia*) is a microbial community proposed to couple CO_2_ fixation to extracellular electron transfer and O_2_ reduction. Previous metagenomic analysis of a single MCL bioelectrochemical system (BES) resulted in resolution of 16 bin genomes. To further resolve bin genomes and compare community composition across replicate MCL BES, we performed shotgun metagenomic and 16S rRNA gene (16S) sequencing at steady‐state current. Clustering pooled reads from replicate BES increased the number of resolved bin genomes to 20, over half of which were > 90% complete. Direct comparison of unassembled metagenomic reads and 16S operational taxonomic units (OTUs) predicted higher community diversity than the assembled/clustered metagenome and the predicted relative abundances did not match. However, when 16S OTUs were mapped to bin genomes and genome abundance was scaled by 16S gene copy number, estimated relative abundance was more similar to metagenomic analysis. The relative abundance of the bin genome representing ‘*Ca*. Tenderia electrophaga’ was correlated with increasing current, further supporting the hypothesis that this organism is the electroautotroph.

## Introduction

Biocathodes are electrodes that have been colonized by one or more microbial species able to mediate the heterogeneous electron transfer reaction from a solid electrode interface to some terminal electron acceptor (Strycharz‐Glaven *et al*., 2013; Schroder *et al*., 2015; Tremblay and Zhang, 2015). Biocathodes have gained increasing attention over the past 5–10 years based on their ability to catalyse the oxygen reduction reaction in microbial fuel cells (MFCs; Ter Heijne *et al*., 2010) and their potential to reduce carbon dioxide into useful products, a process called microbial electrosynthesis (Rabaey and Rozendal, 2010; Lovley and Nevin, 2013). This interest has led to many publications exploring the microbial communities associated with enriched biocathodes including 16S rRNA gene sequencing and clone isolation (Rabaey *et al*., 2008; Huang *et al*., 2011; Pisciotta *et al*., 2012; Rothballer *et al*., 2015; Rowe *et al*., 2015). More recently, some studies have used metagenomic sequencing to resolve the identities of microorganisms associated with biocathodes in an effort to explore their metabolisms *in situ* (Wang *et al*., 2015b; Desmond‐Le Quemener *et al*., 2016; Eddie *et al*., 2017). Despite these efforts, no model biocathode organism has been identified and studied to date with the same success as *Geobacter* has had for anode microbial catalysis (Snider *et al*., 2012; Yates *et al*., 2016; Levar *et al*., 2017). Traditional cultivation approaches may not be appropriate to study biocathode organisms outside of the electrode environment, especially if they require specific growth conditions (e.g. oxygen gradients) that are difficult to reproduce in the laboratory or require partnership with other members of the community. The inability to cultivate interesting biocathode organisms as well as the unknown contribution from the biocathode microbial community as a whole highlights the need to characterize these systems using culture‐independent techniques. DNA sequencing can be used to survey the diversity of these communities *in situ*, which can establish an understanding of key biocathode organisms, underlying community metabolisms, community dynamics and potential interspecies interactions involved in EET and energy conservation.

We previously reported on an aerobically grown biocathode community enriched from seawater and referred to as Biocathode MCL (*Marinobacter*,* Chromatiaceae*,* Labrenzia*). Metaproteomic and metatranscriptomic analysis of MCL revealed expression of proteins related to extracellular electron transfer (EET) and carbon fixation by a previously uncharacterized member of the *Chromatiaceae* family of Gammaproteobacteria (Wang *et al*., 2015b; Eddie *et al*., 2017). This proposed electroautotroph was subsequently assembled to completion using single‐molecule real‐time (SMRT) sequencing reads and putatively identified as ‘*Candidatus* Tenderia electrophaga’ (Eddie *et al*., 2016a). While circumstantial evidence indicates that the majority of heterogenous EET could occur due to growth of ‘*Ca*. electrophaga’ at the electrode surface, we have thus far been unable to provide biochemical evidence for a direct link between cathodic current and the abundance of this organism due to an inability to obtain a pure culture. Additionally, the role and stability of other abundant heterotrophic bacteria at the electrode surface are unknown.

In this study, we further resolve putative MCL bin genomes for ongoing research aimed at exploring the role of ‘*Ca*. Tenderia electrophaga’ and other dominant members of the MCL community in biocathode EET. We present results of metagenomic assembly and clustering using several different computational software packages. Additionally, we compare the utility of filtered/unassembled metagenomic sequencing reads and operational taxonomic units (OTUs) generated from seven primer sets targeting all hypervariable regions of the 16S rRNA gene in assessing the relative abundance using multiple popular computational packages. We confirm that maximum current achieved by each replicate BES is correlated with the relative abundance of uncultured organism ‘*Ca*. Tenderia electrophaga’ based on both metagenomic analysis and 16S OTU analysis. This study provides guidance for using metagenomic and 16S sequencing to characterize low complexity microbial communities that may contain previously uncharacterized microorganisms, such as those associated with biocathodes.

## Results and discussion

### High‐resolution assembly and clustering of Biocathode MCL indicate at least 20 putative bin genomes

Previous metagenomic sequencing of Biocathode MCL indicated at least 16 putative bin genomes. Since this initial study, we have sequenced a total of 10 Biocathode MCL genomes, which are listed in Table S1. ‘*Ca*. Tenderia electrophaga’ (Eddie *et al*., 2016a) and *Anderseniella* sp. were assembled to completion from metagenomic DNA and *Marinobacter* sp. CP1 (Wang *et al*., 2015a) and *Labrenzia* sp. CP4 (Wang *et al*., 2016) were assembled to completion using single‐molecule real‐time (SMRT) sequencing reads. It is important to note again that ‘*Ca*. Tenderia electrophaga’, as well as *Anderseniella* sp., have thus far not been cultivated in isolation. We also sequenced *Marinobacter* sp. CP1 and *Labrenzia* sp. CP4 using the Illumina platform, reported for the first time here. Seven additional MCL bacterial isolates from our freezer collection were sequenced using the Illumina platform. Five of these strains could be mapped back to either a bin genome or 16S amplicon. Details of strain isolation and Illumina sequencing of these seven isolates can be found in the Supplemental Materials and Methods. Taking into consideration this additional genomic sequencing data, our goal in this study was to further resolve reads generated by the Illumina HiSeq platform into metagenomic bins using additional assembly and clustering approaches and compare their relative abundance across replicate MCL reactors grown to maximum current (Table 1). We also assessed the utility of unassembled short metagenomic sequencing reads and 16S sequencing for predicting MCL diversity and relative abundance of taxa. An overview of our computational workflow is presented in Fig. S1.

**Table 1:** Bioelectrochemical system (BES) reactor ID, accession number, metagenomic paired read counts and electrochemical metrics

BES reactor ID	BioSample accession no.	Filtered paired metagenomic reads	Hours to max current	Hours operated before sampling	Max current (i*L*), mA m^−2^	Current at time of sampling (mA m^−2^)
1031813	SAMN04934622	20 069 216	187	256	−9.17	−8.8
2021213	SAMN04934623	23 194 650	178	302	−12.5	−13.4
2031813	SAMN04934624	16 614 934	139	256	−13.6	−13
2040813	SAMN04934625	–	308	344	−37	−44.5
3040813	SAMN04934626	12 545 356	258	344	−42.8	−42
4021213	SAMN04934627	28 163 312	280	303	−23	−23
4032113	SAMN04934628	21 494 123	209	243	−18	−17
4040813	SAMN04934629	17 730 458	165	343	−18	−13

The previously reported metagenome for MCL was generated using Velvet, an assembler not specifically tuned for metagenomic analysis, and MetaWatt, a binning procedure that required a large amount of user intervention (Wang *et al*., 2015b). In this study, two different assembly packages designed to handle metagenomic data, Ray (Boisvert *et al*., 2012) and IDBA‐UD (Peng *et al*., 2012), were compared and used to assemble sequencing reads from seven of the eight BES reported here either individually or after pooling. Read recovery from the 8th BES was insufficient for metagenomic analysis but was used for 16S rRNA gene sequencing analysis below. Ray assembly parameters were optimized based on Illumina sequencing and Ray assembly of *Marinobacter* sp. CP1 and *Labrenzia* sp. CP4 isolates. IDBA‐UD is designed to assemble short sequencing reads when uneven sequencing depth is expected and customization of assembly parameters based on previous optimization of single genome assemblies with even sequencing was not performed. Metrics for all assemblies and clustering approaches can be found in Tables S2 and S3.

First, metagenomic assemblies from individual BES were clustered into putative bin genomes using maxbin (Wu *et al*., 2014), which requires no user input. Relatively few bin genomes were recovered compared with the previously reported MCL assembly (Wang *et al*., 2015b). Between 4 and 10 bin genomes were recovered for each replicate BES using Ray. Attempted taxonomic classification of bins generated from the Ray assembly revealed that many contained housekeeping genes with no close match in the AMPHORA2 database and some had more than one copy of the single copy genes, first noted when the total number exceeded the 31 used for classification (Table S3). These results suggest bin contamination or misassembled contigs, and therefore, relative abundance estimates were not determined. Genomic bins that could be confidently classified always represented organisms previously reported to be among the most abundant. The number of recovered bin genomes increased with the IDBA‐UD assembly, but overall bin completeness and bin contamination did not improve. Taxonomic classification of bin genomes generated from the IDBA‐UD assembly was not attempted based on poor results with Ray. For both Ray and IDBA‐UD, the number of bins recovered from a given BES increased with increasing sequencing coverage (Table S2). The previously reported metagenome for MCL was generated from a sample with a much higher read coverage than was achieved for any sample in this study, which, in addition to a high degree of manual curating, is likely the reason 16 bins could be resolved and classified with confidence from a single sample.

The use of pooled reads from replicate samples provides greater computational power to more accurately cluster assembled reads and leads to fewer misassignments of contigs based on GC content and coverage depth (Albertsen *et al*., 2013). Therefore, reads from all reactors were pooled prior to assembly to improve bin genome recovery. Three different genome clustering packages designed to handle pooled sequencing data from multiple metagenomic samples were assessed: maxbin 2.0, groopm and metabat. The resulting assembly‐clustering cases are denoted in the text first by the assembly platform used, followed by the clustering package (e.g. ray‐maxbin 2.0). Default settings were used for maxbin 2.0 and groopm. Three default settings were assessed for metabat: sensitive (metabat 1), super‐sensitive (metabat 2) and super‐specific (metabat 3).

Regardless of clustering method, both pooled assemblies resulted in a greater number of bin genomes identified per BES, contained a more complete set of housekeeping genes per cluster and initially indicated less potential cluster contamination than had been noted for individual assemblies (Table S3). In general, predicted bin genomes with ≥ 2 Mbps were relatively complete based on comparison with closely related known organisms using CheckM (Table S3). In order to assess clustering accuracy, bin genomes generated from all assembly‐clustering cases were mapped to the 10 available draft MCL genomes using mummer (Table S3). Manual inspection of potentially misassigned contigs (contigs from a single bin genome that map to multiple isolate strains) revealed that a single contig from the IDBA‐UD assembly mapped to the genomes of two different *Flavobacteriaceae* MCL isolate strains (strains 5 and 8). This indicates potential interspecies misassemblies even though by certain metrics the assemblies appeared good (e.g. 45–82 contigs representing 4.26–4.34 Mbp). Additional instances of misassembly were noted within IDBA‐UD bin genomes only (Table S3 IDBA‐UD bins: mbatse24, mbatss27, mbatvs21, groopm071 and mbin11); therefore, IDBA‐UD assemblies were considered unreliable and no further analysis was performed.

Clustering of the Ray assembly using metabat resulted in a higher number of clustered bin genomes than maxbin 2.0 and had lower instances of contig misassignment than both maxbin 2.0 and groopm (Table S3). Therefore, metabat was considered the superior clustering approach in this case and was used for all subsequent comparisons presented in this paper. groopm clustered a similar number of bin genomes as metabat; however, the default setting for groopm clusters shorter sequences (< 2500 bp), which ultimately may have led to the higher number of contig misassignments. Both maxbin 2.0 and groopm have a large number of parameters that can be tuned that may ultimately improve misassignments but were not explored in this study.

**Figure 1:**
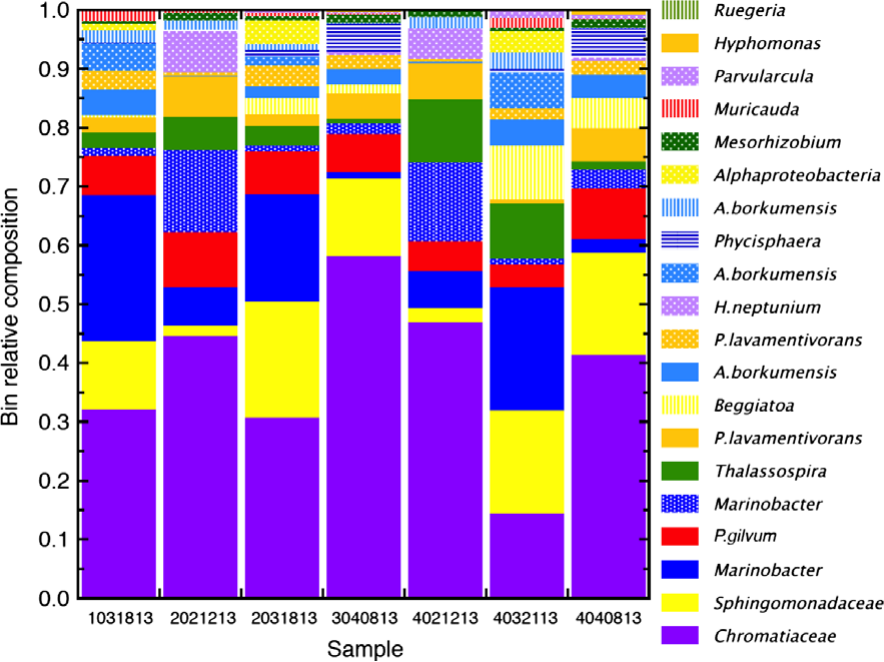
Relative abundance of 20 resolved dominant ray‐metabat case 1b bin genomes to each other classified by AMPHORA2 (i.e. *Sphingomonadaceae* should be *Kordiimonadaceae* as noted in the main text). Unresolved bin genomes are assumed to make up < 1% of the Biocathode MCL community in each reactor as bin genomes shown here make up at least 1% relative abundance. Short contigs that were not binned may belong to identified bin genomes but were not clustered do to cut‐off value. Sample IDs are for each individual bioelectrochemical system (BES).

All clustering approaches, including metabat, resulted in split clusters for the *Marinobacter* sp. CP1 bin genome and several other putative bin genomes identified as *Muricauda*,* Alcanivorax* and *Kordiimonas*. Applying the default MetaBAT parameters for each sensitivity setting always resulted in six predicted *Marinobacter* genomes. Three of these six mapped to different regions of the same complete *Marinobacter* sp. CP1 genome, suggesting that contigs had been inaccurately clustered as opposed to belonging to six independent strains of *Marinobacter*. metabat has an option to use correlation‐based recruiting which is normally off for < 10 samples but was turned on for ray‐metabat with the ‘–sensitive’ case, denoted as ray‐metabat case 1b which did not resolve the split clustering but did further resolve contig assignment to bins. The three bins that mapped to *Marinobacter* sp. CP1 genome were assigned manually to a single bin genome (k61mbatsc03m). The remaining three *Marinobacter* bin genomes were manually assigned to a second *Marinobacter* bin genome based on their similar read coverage and taxonomic identity of housekeeping genes from AMPHORA2 analysis that were not duplicated among the separate bins (k61mbatsc10m). This approach was also used to manually merge the *Muricauda* (k61mbatsc29m), *Alcanivorax* (k61mbatsc22m) and *Kordiimonas* (k61mbatsc02m) split clusters into single genomic bins. Metrics and identities of the 20 final resolved ray‐metabat case 1b bin genomes predicted to be > 55% complete are presented in Table 2, and relative abundance using the AMPHORA2 classification from each replicate BES is depicted graphically in Fig. 1 and Fig. S2. Merged bins are indicated with an ‘m’ following the bin identification. We consider these final resolved bin genomes to be representative of the dominant Biocathode MCL community. Also included in Table 2 are the identities (RDP classification) of the 16S rRNA gene sequences (see below for description) associated with these bin genomes and the associated isolate strain if known. An additional 5 bin genomes were resolved by Ray‐metabat case 1b (Table 2); however, the completeness of these bins was either predicted to be very low or no close genome match was available for comparison. This result suggests that these bin genomes either contain misgrouped contigs or low‐abundance organisms that had too little coverage to generate a sufficient number of contigs.

**Table 2:** Metrics of all ray‐metabat case 1b bin genomes generated from pooled reads of replicate Biocathode MCL bioelectrochemical systems (BES) and associated isolate strain or genome and 16S rRNA gene sequence from in‐house database

Bin genome metrics					Associated strain or genome and long 16S rRNA gene classification			
Bin ID^a^	Classification (AMPHORA2)^b^	Genome size (Mbp)	Number of contigs	Completeness (%)	Isolate strain or genome (Table S1)	Number of contigs mapping to genome	16S rRNA gene RDP classification (in‐house database)	Reference
k61mbatsc09	*Polymorphum gilvum*	5.43	27	86.6	*Labrenzia* sp. CP4	27	*Labrenzia* (g)	Wang *et al*., 2016 Genome A
k61mbatsc01	*Chromatiaceae* (f)	3.71	72	92.26	‘*Ca*. Tenderia electrophaga’	72	*Thiohalobacter* (g)	Eddie *et al*., 2016 IJSEM
k61mbatsc03 m	*Marinobacter hydrocarbonoclasticus* (s)	6.17	217	84.48	*Marinobacter* sp. CP1	187	*Marinobacter* (g)	Wang *et al*., 2015a Genome A
k61mbatsc10 m	*Marinobacter hydrocarbonoclasticus* (s)	6.69	968	55.28	–	455	*Marinobacter* (g)	
k61mbatsc02 m	*Sphingomonadaceae* (f)	4.22	123	95.22	–	–	*Kordiimonas* (g)	
k61mbatsc12	*Thalassospira* (g)	2.71	32	66.83	–	–	*Pannonibacter* (g)	
k61mbatsc21	*Parvibacula lavamentivorans* (s)	3.53	90	90.46	–	–	*Parvibaculum* (g)	
k61mbatsc26	*Alphaproteobacteria* (c)	4.39	402	90.88	*Anderseniella* sp.	398	*Anderseniella* (g)	
k61mbatsc22 m	*Alcanivorax borkumensis* (s)	3.18	64	89.85	–	–	*Alcanivorax* (g)	
k61mbatsc20	*Hyphomonas neptunium* (s)	3.31	67	96.12	–	–	*Hyphomonas* (g)	
k61mbatsc07	*Alkanivorax borkumensis* (s)	3.64	25	98.89	CP2c	16	*Alcanivorax* (g)	
k61mbatsc14	*Beggiatoa_alba* (s)	3.57	89	97.34		–	*Thioprofundum* (g)	
k61mbatsc29 m	*Muricauda ruestringensis* (s)	4.1	383	93.66	CP2a	267	*Muricauda* (g)	
k61mbatsc24	*Phycisphaera* (g)	3.16	712	82.18	–	–	*Phycisphaera* (g)	
k61mbatsc25	*Ruegeria sp. TM1040* (s)	4.25	124	95.76	ND6WE1B	11	*Phaeobacter* (g)	
k61mbatsc11	*Parvibacula lavamentivorans* (s)	3.41	22	85.22	ND6WE1B	14	*16S too short*	
k61mbatsc17	*Alanivorax borkumensis* (s)	3.38	75	95.78	CP2c	74	*16S too short*	
k61mbatsc27	*Mesorhizobium* (g)	4.29	800	85.52	–	–	*16S too short*	
k61mbatsc31	*Parvibacula bermudensis* (s)	2.33	759	62.16	–	–	*16S too short*	
k61mbatsc32	*Hyphomonas* (g)	2.58	787	68.56	ND6WE1B	195	*16S too short*	
k61mbatsc15	*Proteobacteria* (p)	0.26	22	0	–	–	*16S too short*	
k61mbatsc18	*Alphaproteobacteria* (c)	1.01	10	4.17	–	–	*16S too short*	
k61mbatsc19	*Alphaproteobacteria* (c)	0.5	6	0	–	–	*16S too short*	
k61mbatsc08	*No genes found*	0.37	11	0	–	–	*16S too short*	
k61mbatsc28	*Alphaproteobacteria* (c)	0.53	158	2.51	–	–	*16S too short*	

a ‘m’ indicates a merged bin genome.

b g = genus, f = family, s = species, c = class.

In general, the most abundant taxa were similar to those previously reported for Biocathode MCL (Wang *et al*., 2015b). Bin genomes classified by AMPHORA2 as *Chromatiaceae* (further resolved and classified ‘*Ca*. Tenderia electrophaga’), *Marinobacter* and *Polymorphum gilvum* (further resolved and classified as *Labrenzia* sp. CP4) were still among the most abundant, but the bin genome representing *Alcanivorax* sp. made up a much smaller percentage of total read coverage than previously reported. Also, the bin genome classified as the family *Sphingomonadaceae* made up a higher relative abundance than was previously reported. As noted previously (Wang *et al*., 2015b), this bin genome appears to belong to the family *Kordiimonadaceae* rather than *Sphingomonadaceae*, which at the time of this analysis had not been updated in the AMPHORA2 database. For most reactors, the bin genome classified as belonging to the family *Chromatiaceae* was the most highly abundant representing between ca. 30–60% of the community. The one exception was reactor 4032113, which had a higher relative abundance of the bin genome classified as *Marinobacter*.

Pooling multiple samples increased resolution of lower abundance organisms compared with individual BES; however, the lowest abundance organisms could still not be accurately classified. Further increasing the read coverage for Biocathode MCL could improve reliability of classification and abundance estimates of these least represented taxa due to the relative low complexity of the community.

### Unassembled metagenomic reads overpredict diversity of Biocathode MCL compared with assembled, clustered reads

While metagenomic contig assembly and clustering can provide near‐complete bin genomes for the dominant organisms of a microbial community, as shown here this approach is dependent upon the quality and quantity of reads and can be a labour‐intensive process if all that is desired is an estimate of diversity. Classification of unassembled metagenomic reads is an attractive approach to estimating overall diversity of microbial communities, as it does not depend on the quality of contig assembly (Peabody *et al*., 2015). In order to determine how this approach compared with assembled/clustered reads, metaphyler (Liu *et al*., 2011), Kraken (Wood and Salzberg, 2014) and nucleotide–nucleotide blast (blastn) search (Altschul *et al*., 1990) were applied to MCL unassembled reads (Fig. S3A–C respectively). In summary, metaphyler (Fig. S3A) returned assigned reads representing 77 orders, 150 families and 269 genera across all replicate BES (Table S4). Kraken (Fig. S3B) returned 171 orders, 374 families and 800 genera across all replicate BES. Returns using blastn (Fig. S3C) were similar and reported 226 orders, 508 families and 1153 genera across all replicate BES when the entire database was included in the search, i.e. not specific for prokaryotes. These estimates overpredict richness at all three taxonomic ranks compared with assembled and binned contigs with representatives of 35% of the genera listed in the NCBI taxonomy using blastn. All three methods predicted a much lower relative abundance than assembled contigs for the order Chromatiales (5.72%), which contains ‘*Ca*. Tenderia electrophaga’. blastn predicted the highest percentage of hits to the order Chromatiales, which may be due to the fact that this approach utilizes the entire NCBI database.

In order to determine whether relative abundance estimates of the most dominant taxa were similar among the three methods we compared the top 25 orders predicted to be present at > 0.5% by each approach (Table S5). The relative abundance of 10 of these 25 was correlated (*R* ^2^ ≥ 0.78) when results from metaphyler and blastn were compared, while the relative abundance of only five was correlated (*R* ^2^ ≥ 0.78) between metaphyler and kraken (two of which were also correlated between metaphyler and blastn). The relative abundance of only a single order was correlated between kraken and blastn. These results demonstrate that even at higher taxonomic ranks, there is poor agreement between different classification methods when unassembled short reads are used alone and the utility in estimating relative abundance of dominant members of Biocathode MCL with this approach is very limited compared with assembled/clustered contigs.

The estimated increase in community diversity compared with metagenomic assemblies and disagreement in classification between methods may be due to several factors, including: (i) lack of sufficient representation of novel organisms in the database associated with a particular method, (ii) short sequencing reads alone can only be reliably classified at higher taxonomic levels (noted by the high percentage of Proteobacteria that were not classified), and (iii) the metrics used by a given method to assign classification, i.e. marker gene, consensus *k*‐mer, exclude reads from novel organisms. For example, if a novel organism is part of Biocathode MCL and no near match is found in the database specified by a given method, reads may be assigned to the most likely candidate present in that database, unintentionally resulting in a greater number of reads assigned to the wrong taxa or artificially increasing diversity of the community. This may have occurred here for reads associated with ‘*Ca*. Tenderia electrophaga’, which does not fit cleanly into either the Chromatiales or Thiotrichales (Eddie *et al*., 2016a).

### Classification of OTUs from 16S rRNA gene sequencing is consistent with the metagenomic sequencing

MCL community diversity was estimated by 16S amplicon sequencing of all variable regions from all eight replicate MCL BES (Table S6). OTUs were generated and classified using both CD‐HIT with RDP classification (CD‐HIT/RDP) and the mothur workflow (Table S7); mothur consistently predicted a greater diversity of bacteria than CD‐HIT/RDP, identifying between 64 and 440 MCL OTUs for a given variable region. CD‐HIT/RDP predicted between 29 and 69 OTUs depending on the variable region. This may be due to the fact that CD‐HIT excludes OTUs based on a number of parameters built into the analysis while mothur allows all possible OTUs with at least two reads to be retained. Due to longer amplicon length, the V7/8 region produced fewer paired reads than other regions; therefore, while relative abundance counts were calculated for this region, the predicted number of species may not represent the MCL community as accurately as others.

In general, estimated relative abundance of MCL taxa agreed very well between CD‐HIT/RDP and mothur (Fig. S4A and B). Dominant predicted taxa among both methods for most variable regions included the orders Alteromonadales, Oceanospirillales, Chromatiales, Rhodobacterales and Kordiimonadales, generally consistent with those identified by assembled/clustered metagenomic sequences and unassembled reads. However, where all other CD‐HIT/RDP OTU assignments agreed with mothur, OTUs from the V5 region were classified as being of the order Pseudomonadales rather than Chromatiales. In addition, < 0.1% of CD‐HIT/RDP OTUs from the V1/2 and V6 regions were classified as being of the order Chromatiales and were instead assigned to Oceanospirillales. Drawing from previous taxonomic analysis of Biocathode MCL generated by our group, we decided to pursue orthogonal validation of the classification of these OTUs.

Operational taxonomic units generated by CD‐HIT were aligned with a previously generated in‐house library of 22 longer MCL 16S sequences recovered from a progenitor MCL BES (Strycharz‐Glaven *et al*., 2013), MCL strain genomes and metagenomic contigs from both the Ray and IDBA‐UD assemblies and classified using RDP (Table 2 and Table S8). OTUs that mapped to the in‐house 16S library accounted for 97–99% of all reads for every variable region across all BES except the V1/2 region (Table S8). This may be due to difficulties in mapping reads accurately if the longer sequence was truncated in this end region of the gene. In another instance, > 99% of reads from the V1/2 region of BES 4021213 were not mapped to a longer sequence indicating some technical problem during amplification of this sample. Longer 16S sequences from the in‐house library could be mapped to 14 of 20 bin genomes (*Marinobacter* spp. are combined) and the associated OTUs accounted for 90–95% of all 16S sequencing reads (Table S9), further demonstrating that bin genomes resolved in this study represent the majority of MCL constituents. OTUs that could not be mapped to a longer 16S sequence likely belong to rare members of the community.

When CD‐HIT OTUs from the V1/2, V5 and V6 regions were now linked to longer 16S sequences, they were classified to the order Chromatiales, bringing relative abundance estimates into closer agreement with the mothur workflow. This indicates that the order containing the newly described ‘*Ca*. Tenderia electrophaga’ may not be classified correctly using CD‐HIT/RDP depending on which variable region is sequenced and has implications for studies using this workflow where amplicons cannot be validated against longer sequences.

**Figure 2:**
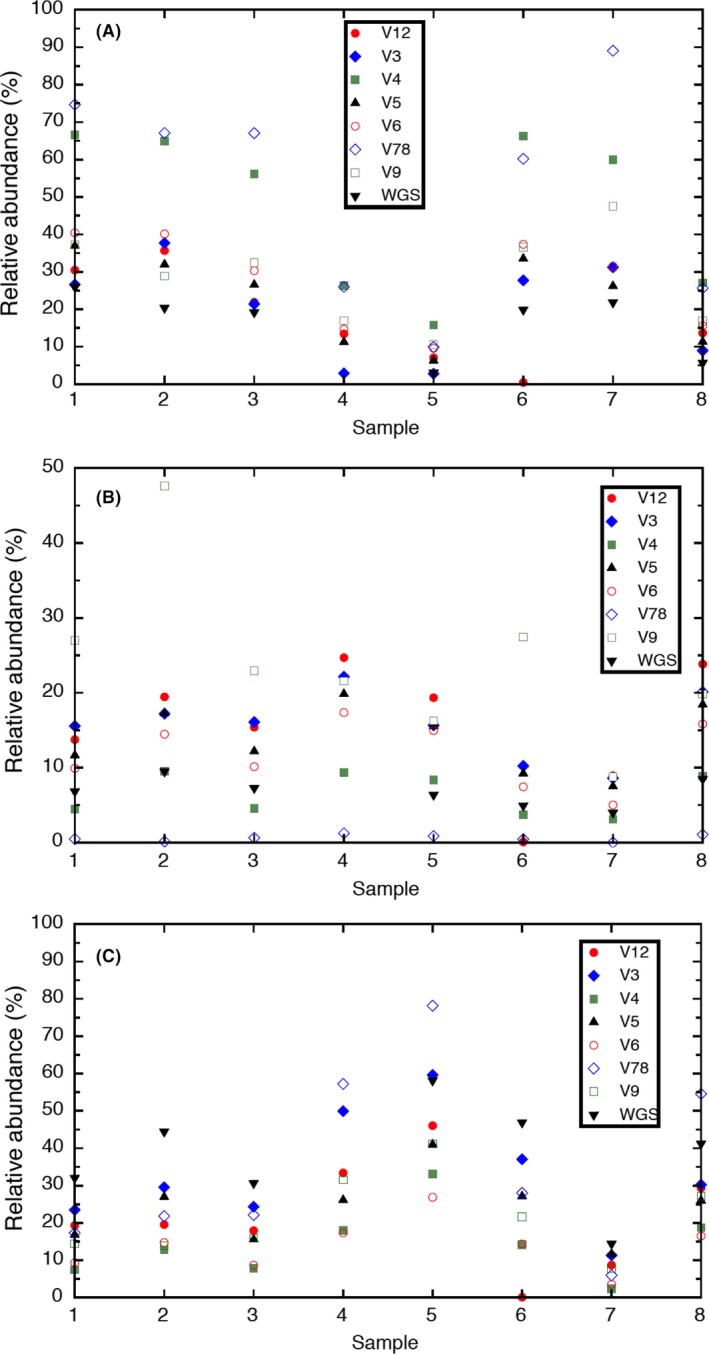
Relative abundance estimated for bin genomes from each replicate bioelectrochemical system (BES) representing (A) *Marinobacter* spp., (B) *Labrenzia* sp. and (C) ‘*Ca*. Tenderia electrophaga’ using read counts associated with OTUs linked to longer 16S sequences or generated by whole‐genome shotgun (WGS) sequencing and resolved by ray‐metabat case 1b (BES key: 1 = 1031813, 2 = 2021213, 3 = 2031813, 5 = 3042313, 6 = 4021213, 7 = 4032113, 8 = 4040813).

Despite linking to longer 16S reads, OTU read counts fluctuated between individual variable regions (Table S5), and which variable regions were in agreement differed by bin genome. For example, the V5 and V6 regions of the *Marinobacter* bin genome were always within 5% relative abundance of each other for all BES; however, V4 differed by more than 20% (Fig. 2A). At the same time, V3 and V5 were in better agreement for the *Labrenzia* bin genome (Fig. 2B) and V4 and V6 for the ‘*Ca*. Tenderia electrophaga’ (Fig. 2C) for all BES. As noted in other studies looking at 16S amplicon sequencing (Peiffer *et al*., 2013; Pascual *et al*., 2016), these observations may indicate primer bias in amplification of certain variable regions between organisms or variability in the degree of conservation between regions. Knowing ahead of time how relative abundance is influenced by variable region is useful when 16S sequencing is used alone to gauge community diversity (Yarza *et al*., 2014). However, this information may not be known *a priori* unless longer 16S sequences are available, as was the case here. In cases where accuracy of variable regions cannot be assessed independently, we conclude that as long as the same variable region is used consistently for a given community (e.g. V3 is always used for MCL), general predictions between samples are at least qualitatively accurate without genomic validation and no single variable region is necessarily more appropriate. Sequencing all variable regions provides the best buffer against these fluctuations because read counts can be averaged across all variable regions.

### Relative abundance of dominant bin genomes is comparable between metagenomic and 16S estimates after scaling for genome abundance

The average relative abundance across all variable regions for each BES was calculated for OTUs that could be linked to longer 16S sequences and mapped to bin genomes (Table S8). This provided an average estimate of relative abundance for the majority of bin genomes based on 16S sequencing. In order to relate these values to relative abundance of bin genomes predicted from ray‐metabat case 1b, genome abundance was scaled to 16S estimates using the known or predicted copy number of the 16S rRNA gene (Table S8). Abundance estimates for the two predicted *Marinobacter* subclusters were summed in order to account for the fact that a single 16S sequence represents both. The fact that only a single 16S sequence was identified for two predicted bin genomes is not unexpected as they likely have 16S rRNA genes too similar to generate separate OTUs. Relative abundance of each bin genome after scaling was generally in agreement with that predicted by linked CD‐HIT OTUs (Table S8).

### Relative abundance of ‘*Ca*. Tenderia electrophaga’ is correlated with relative abundance

**Figure 3:**
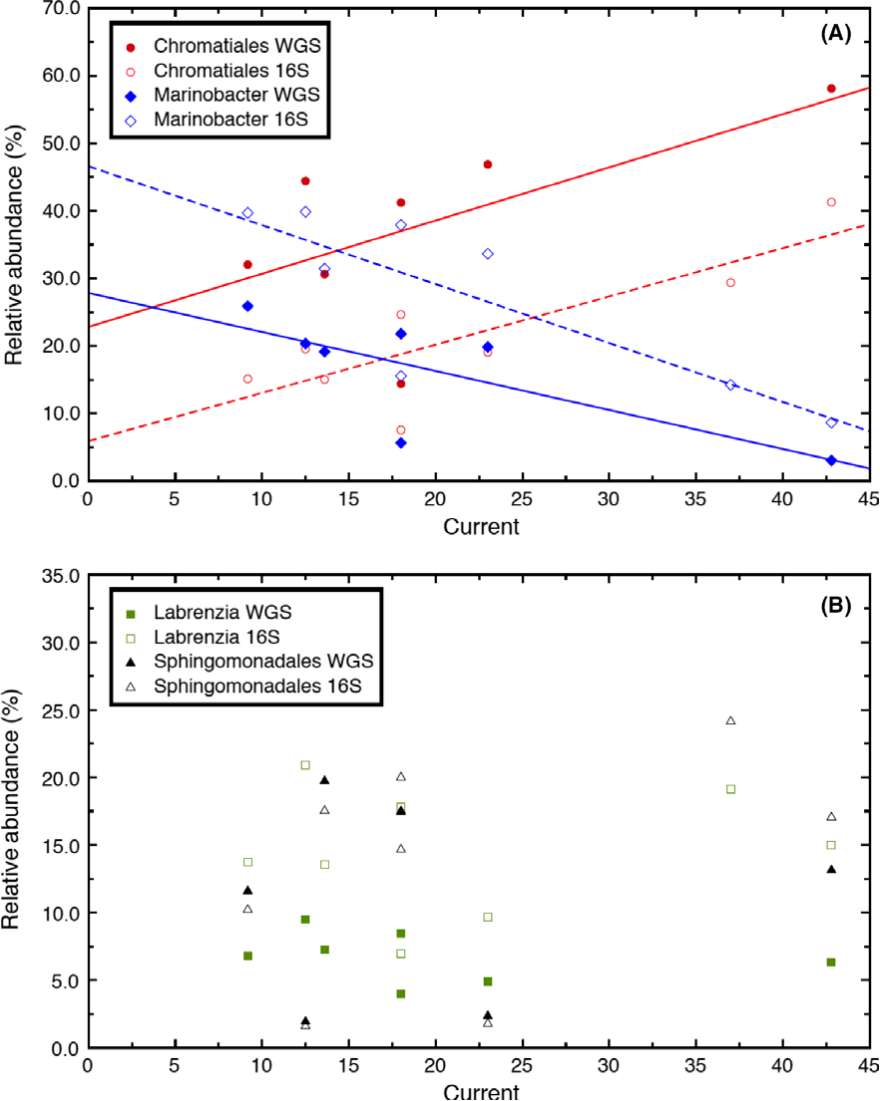
Correlation of per cent abundance of (A) *Marinobacter* spp. (combined bin genomes) and the order Chromatiales (representing ‘*Ca*. Tenderia electrophaga’) to absolute current, as well as (B) *Labrenzia* sp. and the order Sphingomonadales (Kordiimonadales) using read counts associated with OTUs linked to longer 16S sequences or generated by whole‐genome shotgun (WGS) sequencing and resolved by ray‐metabat case 1b (BES key: 1 = 1031813, 2 = 2021213, 3 = 2031813, 5 = 3042313, 6 = 4021213, 7 = 4032113, 8 = 4040813).

Current measured using a potentiostat (chronoamperometry) provides a real‐time estimate of MCL microbial activity at the electrode surface and approximates growth (by observation of cathodic current increasing over time). In order to determine whether a relationship exists between increasing cathodic current and the presence of dominant members of the MCL community, we compared both relative abundance predicted from averaged 16S OTU read counts and ray‐metabat case 1b bin genomes to the recorded maximum current output for each replicate BES at the time of sampling (Table 1). Relative abundance determined by either method showed that the order Chromatiales*,* which represents *‘Ca*. Tenderia electrophaga’, was positively correlated to current (*R* ^2^ 0.627–0.874, *P*‐value 0.034 for *n* = 8) (Fig. 3A). *Marinobacter* was negatively correlated with increasing cathodic current (*R* ^2^ 0.666–0.798) and there was no correlation between current and any other bin genomes or OTU, including those representing *Labrenzia* and *Kordiimonas* (Fig. 3B). *Marinobacter* spp. are known to oxidize iron and the observation that they may be detrimental to cathodic current here is an observation that should be further explored. We do not rule out the possibility that other MCL constituents are somehow associated with the capacity of MCL for extracellular electron transfer resulting in negative current; however, with the limited number of samples included in this study, we cannot make further inference at this time. It is possible that some of these organisms play a role in allowing for the growth and maintenance of ‘Ca. Tenderia electrophaga’ on the electrode by engaging in syntrophic relationships. Whether only some or all are needed is a research question currently being examined.

## Conclusions and implications

Results presented here show that while some degree of variability in relative abundance exists between replicate MCL BES, the most abundant constituents remain the same. A clear positive correlation existed between ‘*Ca*. Tenderia electrophaga’ and magnitude of cathodic current, which is consistent with this organism's role as the primary electroautotroph. Overall, assembled and clustered metagenomic contigs from pooled reads provided a good estimate of relative species abundance for organisms that are at > 1% of the Biocathode MCL community, resulting in 20 highly resolved bin genomes and improving our previous analysis. Taxonomic classification of unassembled metagenomic sequencing reads largely missed or misassigned the proposed electroautotroph ‘*Ca*. Tenderia electrophaga’ and this approach was not found to be useful for estimating the diversity or the relative abundance of MCL. 16S rRNA gene sequencing of all variable regions resulted in OTUs that were classified to many of the same taxa as bin genomes and assignments were improved for the order *Chromatiaceae* after linking to longer 16S sequences. The potential for misclassification of OTUs representing the recently described electroautotroph, ‘*Ca*. Tenderia electrophaga’, by certain variable regions using the RDP classifier suggests that multiple regions should be chosen when uncharacterized organisms to buffer against this issue.

We recommend that for low complexity biofilms that are highly enriched (i.e. expect similar organisms in each reactor) and may contain previously uncharacterized organisms, an initial metagenomics survey should be performed prior to, or at the same time as 16S analysis, with either (i) sufficiently high read coverage on individual samples which may require significant manual curating, or (ii) multiple samples which may allow for use of binning methods with less user intervention while maintaining accuracy. Metagenomic data should be assembled using multiple assemblers as no one outperforms the others on all fronts and each may have different strengths. Ray may be useful to include as it gave more complete 16S assemblies; however, IDBA‐UD produced many more contigs. This may be very important in communities with greater diversity than MCL. Bin genomes can be used to assign OTUs generated from 16S rRNA gene sequencing to a specific organism, provided contigs contain complete 16S sequences, which potentially improves their utility for monitoring relative abundance by more accurately reflecting the taxonomy of the community. The sequencing analysis tools described here for use with MCL can be used to accurately predict intentional changes to MCL community composition in future studies and could also be useful to characterize other similarly enriched BES‐associated microbial communities.

## Experimental procedures

### Biocathode biofilm cultivation

Bioelectrochemical reactors, artificial seawater medium and growth conditions were identical to those previously reported (Strycharz‐Glaven *et al*., 2013; Leary *et al*., 2015; Wang *et al*., 2015b) and described briefly here and in the Supplemental Materials and Methods. Working electrodes were graphite coupons (length 10.0 cm, width 3.0 cm, height 0.2 cm; total geometric surface area of 65.2 cm^2^ or 0.00652 m^2^). Scrapings from previously enriched biocathode biofilms were used as an inoculum (Strycharz‐Glaven *et al*., 2013; Leary *et al*., 2015; Wang *et al*., 2015b). Working electrodes were maintained at 0.310 V vs. standard hydrogen electrode (SHE; 0.100 V vs. 3M KCl Ag/AgCl) using a multichannel potentiostat (Solartron 1470E) under software control (multistat v1.6d, Scribner Associates Inc., Southern Pines, NC, USA). Once maximum steady‐state current was achieved (values listed in Table 1), biocathode biofilm DNA was collected by scraping the electrode surface with a fresh, sterile razor blade and scrapings were used directly in the DNA extraction protocol described below.

### Metagenomic DNA sequencing and processing

Metagenomic DNA for whole‐genome shotgun (WGS) sequencing was extracted from all eight graphite coupon electrodes using the MoBio PowerBiofilm^®^ DNA Isolation Kit. DNA integrity and concentration were verified using a Bioanalyzer 2100 (Agilent, Palo Alto, CA, USA) and Qubit^®^ dsDNA HS assay kit (Life Technologies, Grand Island, NY, USA) and sequenced on an Illumina HiSeq 2000 sequencer using the TruSeq SBS Kit v3‐HS sequencing kit (Life Technologies, Grand Island, NY, USA) at the Genomics Resource Core Facility at Weill Cornell Medical College (New York, NY, USA). Raw sequence reads were first trimmed using sickle v1.33 (Joshi *et al*., 2011) using default settings and then error‐corrected using Ecc set to 30 of the bbmap utility v34.86 (Bushnell, 2016).

### Metagenome assembly and analysis

Filtered reads from seven of the eight replicate BES were assembled using both Ray v2.3.1(Boisvert *et al*., 2012) (*k*mer length = 61) and idba‐ud v1.1.1(Peng *et al*., 2012) individually and after pooling. Ray and IDBA‐UD were selected based on availability, ease and readiness for implementation and ability to simultaneously analyse replicate metagenomes. We were unable to generate metagenomic sequencing reads from one BES (2040813) due to a library preparation processing failure; however, we did obtain 16S amplicons. Ray settings for *k*‐mer length and node coverage of 8 were selected based on previous Ray assemblies of Illumina *de novo* sequencing of *Marinobacter* sp. strain CP1 (Wang *et al*., 2015a) and *Labrenzia* sp. strain CP4 (Wang *et al*., 2016) as described in the Supplemental Materials and Methods. IDBA‐UD does not allow for customized assembly parameters; therefore, default settings were used. Open reading frames (ORFs) were predicted from assembled contigs using prodigal v2.6.2 (Hyatt *et al*., 2012) prior to clustering.

When reads from replicate BES were assembled separately, contigs > 1 Kbp were clustered into genomic bins using maxbin v1(Wu *et al*., 2014), which can only process a single sample. For pooled read assemblies, maxbin 2.0, metabat v0.25.4(Kang *et al*., 2015) and groopm v0.3.1(Imelfort *et al*., 2014) were used to cluster contigs into bin genomes. This analysis resulted in many solutions, which have been termed assembly‐clustering cases, and denoted in the text first by the assembly platform used, followed by the clustering package (e.g. ray‐maxbin 2.0). Default settings were used for maxbin 2.0 and groopm. Three settings were assessed for metabat: sensitive (metabat 1), super‐sensitive (metabat 2) and super‐specific (metabat 3). Only default or easily implemented optional settings were tested. While groopm and metabat both have many parameters that can be adjusted only metabat provides batch choice settings that set all the parameters at the time of this manuscript preparation. AMPHORA2 was used to classify bin genomes, and checkm v1.05 and mummer v3.23 alignments of bins to Biocathode MCL isolate strains and genomes were used to check bin quality (Supplemental Materials and Methods). The average coverage depth of bin genomes for each BES was determined by mapping the unassembled reads from each sample to metagenome contigs generated from pooled read analysis using bowtie v0.9.6 (Langmead *et al*., 2009). Coverage depth was used to calculate relative abundance of metagenomic bins.

### Unassembled read analysis

Filtered unassembled metagenomic reads for each sample were analysed using blastn (Camacho *et al*., 2009), kraken v0.10.5‐beta(Wood and Salzberg, 2014) and metaphyler v1.25 (Liu *et al*., 2011). blastn analysis was performed by reporting the top five hits for each sequence from the entire NCBI database. An expectation cut‐off score of 0.001 was used. Blastn returns for paired raw reads were examined to generate a final taxonomy score. First, the scores of the next best hits within a read were compared. If the next best hit's *e*‐value was within two orders of magnitude of the top hit, then the taxonomy of the sequence was the common taxonomy. For all paired reads that both had taxonomic returns, the taxonomy shared in common between paired reads was the assigned taxonomy. Counts were based on the number of read counts clustered. kraken and metaphyler were used with their default settings directly on the unassembled sequences. Relative abundance was calculated for each method at each taxonomic rank reported (genus, family or order) as the proportion of reads representing each taxa identified with no cut‐off, i.e. all identified taxa are reported.

### 16S rRNA gene sequencing and analysis

The V1 – 9 regions of the 16S rRNA gene were amplified using a two‐step PCR amplification approach recommended by Illumina. Two adapters (forward 5′‐TCG TCG GCA GCG TCA GAT GTG TAT AAG AGA CAG, reverse 3′‐GTC TCG TGG GCT CGG AGA TGT GTA TAA GAG ACA G) were added to previously published primers for each region (Table S10). The first PCRs were carried out as previously reported (Eddie *et al*., 2017) in a 25 μl of reaction volume using 1 ng genomic DNA as template. The second PCR with index primers was carried out using 10 μl of first PCR products as template with 5 μl each of Illumina index primers in a 50 μl of reaction volume. PCR was performed with initial incubation at 72°C for 3 min, followed by 10 cycles of 98°C, 10″, 63°C, 30″, 72°C, 30″. Microbial 16S rRNA gene sequences of the variable regions of the 16S rRNA gene were acquired on a MiSEQ™ instrument under automated software control (v2.2.0, Illumina, San Diego, CA, USA). Sequences from all samples were pooled together to increase the resolution of low‐abundance OTUs and were processed using default parameters for cd‐hit v4.5.5 (Li *et al*., 2012), and mothur v1.33.3 (Schloss *et al*., 2009). In the case of CD‐HIT, the resulting OTUs were assigned taxonomy using the rdp classifier v2.7 (Wang *et al*., 2007) with 50% confidence setting. At the time of this analysis, the mothur pipeline used the silva v119 reference alignment and the rdp training set v10. Relative abundance was calculated by dividing total read counts associated with each OTU by the total read generated from each replicate BES.

### Comparing abundances from metagenome bins and 16S OTUs

Operational taxonomic units (CD‐HIT) generated from all 16S variable regions were aligned (blast) to a reference database of longer MCL 16S rDNA sequences (97% identity) (Supplemental Materials and Methods). OTUs aligning with the same long 16S sequence were considered linked to each other. Fourteen of these long sequences could be mapped to bin genomes and were used to compare relative abundance estimates based on bin coverage to 16S read counts. The 16S rRNA gene copy number of the *Marinobacter* and *Labrenzia* bin genomes was known based on the closed draft genomes for these organisms. Using these numbers and an initial value of 1 copy for all other bin genomes as scaling factors, we compared genome relative abundance to relative abundance of bin genomes based on linked 16S OTU read counts. Each bin is multiplied by its scaling factor, and then, each new value is divided by the sum of all new values to again express a relative abundance. This comparison was improved by iteratively changing the 16S rRNA gene copy numbers for bin genomes with unknown number of 16S rRNA genes to improve agreement of 16S read count estimates to genomic relative abundance. These estimates were partially validated by comparing with the known 16S gene copy number from the ‘*Ca*. Tenderia electrophaga’ and *Anderseniella* sp. genomes, which were not used initially as scaling factors.

### Accession number(s)

All sequences produced in this study are available in the NCBI Sequence Read Archive under accession number SRP043535 under Bioproject number: PRJNA244670.

## Conflict of interest

None declared.

## Supporting information


**Fig. S1.** Computational workflow overview.

**Fig. S2 (Krona plot, download file before viewing in browser).** Interactive Krona plots depicting relative abundance of 20 resolved dominant Ray‐MetaBAT case 1b bin genomes to each other classified by AMPHORA2 (i.e. Sphingomonadaceae should be Kordiimonadaceae as noted in the main text).

**Fig. S3a‐c (Krona plot, download file before viewing in browser).** Interactive Krona plots depicting relative abundance of predicted taxa at the genus, family, and order levels resolved using Metaphyler (a), Kraken (b), and blastn (c).

**Fig. S4a‐b (Krona plot, download file before viewing in browser).** Interactive Krona plots depicting relative abundance predicted by 16S rRNA gene amplicon sequencing of each hypervariable region for eight replicate bioelectrochemical systems (BES) using OTUs generated by CD‐HIT with RDP classifier (a) or mothur (b).

**Data S1.** Supplemental Materials and Methods.
**Table S1.** Biocathode MCL strain IDs and associated metrics.
**Table S2.** Biocathode MCL metagenomic assembly metrics for both individual and pooled bioelectrochemical system (BES) sample reads.
**Table S3.** Metagenomic binning and quality checks.
**Table S4.** Relative abundance for all 7 replicate bioelectrochemical systems (BES) for which metagenomic reads were generated using Metaphyler, Kraken, and blastn at the genus, family, and order levels.
**Table S5.** Correlation analysis comparing Metaphyler, Kraken and blastn analysis to each other or to current for the top 25 orders identified across all samples (pooled) representing at least 0.5% of total abundance (“‐” indicates at least one method had no counts in the specified order level group).
**Table S6.** Number of read pairs generated for each 16S rRNA gene hypervariable region for each bioelectrochemical systems (BES).
**Table S7.** OTUs found for each 16S rRNA gene hypervariable region using either CD‐HIT or mothur.
**Table S8.** Relative abundance predicted by 16S rRNA gene amplicon sequencing of each hypervariable region for eight replicate bioelectrochemical systems (BES) using OTUs generated by CD‐HIT with RDP classifier.
**Table S9.** Relative abundance estimates of the 20 most dominant bin genomes based on metagenomic sequencing and 16S rRNA gene sequencing.
**Table S10.** Primer sequences for 16S rRNA gene hypervariable regions used in this study.
