# Supplementary material for: Relative abundance of ‘Candidatus Tenderia electrophaga’ is linked to cathodic current in an aerobic biocathode community
Source: Microb Biotechnol. 2017 Jul 11;11(1):98–111. doi: 10.1111/1751-7915.12757 (PMC5743799; doi:10.1111/1751-7915.12757)
Supplement: Supplementary file 2 — Fig. S2 (Krona plot, download file before viewing in browser). Interactive Krona plots depicting relative abundance of 20 resolved dominant Ray‐MetaBAT case 1b bin genomes to each other classified by AMPHORA2 (i.e. Sphingomonadaceae should be Kordiimonadaceae as noted in the main text). [file MBT2-11-98-s002.html]

Javascript must be enabled to view this page.

richness


CH1031813
CH2021213
CH2031813
CH3040813
CH4040813
CH4021213
CH4032113

753.55843.96596.53514.99698.141045.14811.88

12.922.183.620.391.550.7513.66

12.922.183.620.391.550.7513.66

12.922.183.620.391.550.7513.66

12.922.183.620.391.550.7513.66

12.922.183.620.391.550.7513.66

12.922.183.620.391.550.7513.66

739.98838.74586.85490.22663.071044.17793.91

216.44277.27245.85152.56270.39323.2315.57

55.9372.6260.641.7464.2779.4154.58

8.690.0123.810.010.010.0129.6

8.690.0123.810.010.010.0129.6

8.690.0123.810.010.010.0129.6

47.2472.6136.7941.7364.2679.424.98

3.1510.273.997.418.949.774.93

44.0962.3432.834.3255.3269.6320.05

44.0962.3432.834.3255.3269.6320.05

0.7761.571.124.8110.7855.172.02

0.7761.571.124.8110.7855.172.02

0.7761.571.124.8110.7855.172.02

0.1759.170.132.84.6552.970.17

20.146.5420.533.879.87112.5175.42

20.146.5420.533.879.87112.5175.42

20.146.5420.533.879.87112.5175.42

0.830.162.481.714.560.169.03

0.830.162.481.714.560.169.03

0.830.162.481.714.560.169.03

0.830.162.481.714.560.169.03

87.4216.36117.7867.66121.8424.6142.2

87.4216.36117.7867.66121.8424.6142.2

51.3980.0243.3432.7759.0751.3532.32

51.3980.0243.3432.7759.0751.3532.32

51.3880.0243.3232.7759.0651.3532.3

51.3880.0243.3232.7759.0651.3532.3

523.54561.47341337.66392.68720.97478.34

195.31171.94114.3115.8239.66207.67177.14

195.31171.94114.3115.8239.66207.67177.14

195.31171.94114.3115.8239.66207.67177.14

195.31171.94114.3115.8239.66207.67177.14

3.220.0216.368.0236.560.0274.05

3.220.0216.368.0236.560.0274.05

3.220.0216.368.0236.560.0274.05

3.220.0216.368.0236.560.0274.05

241.49375.01182.74299.27287.75489.85117.02

241.49375.01182.74299.27287.75489.85117.02

83.5214.527.5914.5528.7123.43110.13

83.5214.527.5914.5528.7123.43110.13

83.5214.527.5914.5528.7123.43110.13

83.5214.527.5914.5528.7123.43110.13

0.653.046.0624.3833.520.224.31

0.653.046.0624.3833.520.224.31

0.653.046.0624.3833.520.224.31

0.653.046.0624.3833.520.224.31

0.653.046.0624.3833.520.224.31

0.653.046.0624.3833.520.224.31
