# Supplementary material for: Relative abundance of ‘Candidatus Tenderia electrophaga’ is linked to cathodic current in an aerobic biocathode community
Source: Microb Biotechnol. 2017 Jul 11;11(1):98–111. doi: 10.1111/1751-7915.12757 (PMC5743799; doi:10.1111/1751-7915.12757)
Supplement: Supplementary file 3 — Fig. S3a‐c (Krona plot, download file before viewing in browser). Interactive Krona plots depicting relative abundance of predicted taxa at the genus, family, and order levels resolved using Metaphyler (a), Kraken (b), and blastn (c). [file MBT2-11-98-s003.html]

Javascript must be enabled to view this page.

richness


1031813
2021213
2031813
3040813
4040813
4021213
4032113

69720803415884429813496259090771514

144183199188333180357

6231722411440

6231722411440

6231722411440

6231722411440

12152418421436

126145158148250152281

122143143138217144242

42151033839

42151033839

42151033838

7791502435815358940

236801116

236801116

5991191754211748713

48096138339936563

39270118298926444

10111209

50201011

68720798705828629420489599051870030

22543316222571314826240763021923724

61776869727049111701000

252330277106209454357

16259343326

232269234106175417371

3145237129092

21182817211628

299250431208327366507

169114231114182189288

359321517

6387623068614341608371806550

661410853

661410853

61124665370163103

29284423272144

19107911743103714247991190

10314211011114812995

52661012222

200236238200259230274

1224136151012

3652634

14171220121818

13222810629

718076397210489

718076397210489

252303315197253336316

14511812148

12510812148

2210202222510

39571043

1051039716

188392267137200523356

188392267137200523356

860169187512

3381204

103371671511298641911294120278228

51011917711662861124495

935414471984559441048098887167

513166152312

0884931

1182811569

151137435139

10240409

1182416

71261155462014504776474254654

40269411802732310111186075177139436

353827393343

353827393343

2858433036202214522106734097726753

92129869110015965

92129869110015965

2813032494198524309103894031626356

2760031969194614187101963966525880

141091001133

169179160132162256155

169179160132162256155

339262268224306320432

339262268224306319432

27479531067753126914123155

302623348

18124896234057647622174

13263594502895875531601

13651152895818106614631316

13241128869796103714441257

748631468443563791735

7013806

130338014

130338014

434414343298425553525

270270229187267359297

699853566711540

699853556711339

1943234414671489190828012045

7711657878914545

7711657878914545

538541596210331

970110069770689413571052

134602916

171206136133156261160

961649810612120866

0004000

1361649110912521795

315910161337

315910161237

14122411311717426784

14022211211617326483

19481315264811

51043457

51043457

170209206179272249331

1022251114937

902154037

902154036

127155114125155212121

82115778710214151

82115778710214151

7610671829412949

21154268

2444257

2444257

147243701

147243701

147243701

147243701

032121604

032121604

032121604

032121604

265661314759130

4296882414

013133161

013133161

416555813

3840039

74416166103

113003287

102802081

913613141610

913613141610

91361313169

853121268

105815192620

65412182615

65412182615

443392315

0014300

37653452386425

37653452386425

35563248345822

35563248345822

35563248345822

02191617
