# Supplementary material for: Relative abundance of ‘Candidatus Tenderia electrophaga’ is linked to cathodic current in an aerobic biocathode community
Source: Microb Biotechnol. 2017 Jul 11;11(1):98–111. doi: 10.1111/1751-7915.12757 (PMC5743799; doi:10.1111/1751-7915.12757)
Supplement: Supplementary file 4 [file MBT2-11-98-s004.html]

Javascript must be enabled to view this page.

richness


1031813
2021213
2031813
3040813
4040813
4021213
4032113

57979113383639620162623189364353232158624629038648

3170214611997316268214032814127703

1151086695822105013161011

1151086695822105013161011

1151086695822105013161011

103106065678510031288917

50375295341453452466

20308182284282374128

42629312365447781386

10387206313309581155

247253515841749197528321678

69712450516589900643

66711449513582887639

52612382456491762544

52612382456491762544

25420231361314580305

109838598524654898576

77628449451513646404

77628449451513646404

77628449451513646404

77628449451513646404

31792421625621879315

22767350587541851195

22767350587541851195

55194913261932609731122274724233246157157638887497

35496412140964516489215283603267999144251337895274

309657637155163534383153592

309657637155163534383153592

309657637155163534383153592

10219401022145014292312906

114200312121497172825901303

6919841094159615872570899

276583954994953642487117007

262577253834869628786046679

167308635212764378444924437

89103122971105199915633025

527675476979001123949

56185210601384163828181194

23564545475553882734

23564545475553882734

58186810491447165027951121

3910735938698891539602

3910735938698891539602

12366197270294452271

197954565787611256519

197954565787611256519

197954565787611256519

191310022022355284845902503

191310022022355284845902503

181283320722130262942072380

157293925825921257241

13566279441433775172

36474343344426597430

6821122887687129651910076915791967062

1854376192081729373303874950119794

209437226853294384562933129

317313344776021148377

619886337979671481757

76156110851232138521901205

4537983454364236533105384324

4537983454364236533105384324

54111316615787128865143265417

54111316615787128865143265417

6041301169521023310321171936182

6041301169521023310321171936182

92220511591666176727461248

92220511591666176727461248

92220511591666176727461248

48078071745546637046675310262344996

438552533534220459870683482

438552533534220459870683482

10281742398581404814476218649471

10281742398581404814476218649471

2926506902809039928416266424127607

469742040975738616690124065

1773318951751425413263744087417087

63810167582378548152127935726

350600335844605516980253711

159293416752288254741291788

186285117982145237635781795

1931218501414017447197712863217542

1931218501414017447197712863217542

113113817878711013122891777810273

218309719322442264039032348

241251317121994229634022049

519665840465381594882924482

732693147605596656594456619

732693147605596656594456619

46693330481548910364

46693330481548910364

69910765676584119637137658046

69910765676584119637137658046

991129860840111514981243

991129860840111514981243

145200214701543188823761999

145200214701543188823761999

447726042445750632393774532

447726042445750632393774532

4636467533029836126408886043837049

4636467533029836126408886043837049

136914067903410676122041831511190

164283915792141220737281378

169180412621395162621351566

302275416242039244138512200

10614688641264138719071074

40543246366338707294

318535129444111430470132931

318535129444111430470132931

25285162244304372222

25285162244304372222

109210041686475908415132148546

497488033893732420864524031

563498733613708404265364256

893714047175272636490576694

893714047175272636490576694

597514837714463513561514470

597514837714463513561514470

28970511610637477378911170862773127137157377297601

444477243053837466071565668

444477243053837466071565668

10510911111874112015711493

18549427381395739449

84544397433511789621

296033654425141017404

296033654425141017404

402309326002458293744643835

402309326002458293744643835

402309326002458293744643835

13862593051783410571433

13862593051783410571433

627915615035341104840

627915615035341104840

627915615035341104840

28842211597048475935211088242762932136925617277034

60436282363380653439

60436282363380653439

190329346679111163537779

186325545178910963418735

28812811592041475800411075152760945136860327274774

135661527022748012702364617156606420979

27316711410218445717510763512687009134846726813958

5332553670214522309452810350

18610571396988170714633025

19793152977115177117499138807200695169408

19680152055114497116881137980199170168110

219247216401957222434912350

219247216401957222434912350

922650449404868567981717572

922650449404868567981717572

18260141033106357108504128266184880155924

188183312021235143026121795

274252715011983225730862180

294189219651544197324842889

645442636223390393459355492

258213017841564200929762487

177913458104029585116031661015193

269193916941494199126442380

157614375973911630128261924512935

1096767855745686710095918763

29612081198864138916612520

988673954485524632388578079

875605744294457531879276688

426350624582884333242883200

633634540905041560483715314

2229191232794130610812342

194614710995111130129601876214977

2629217061759416174190602907025094

325376024442890328151763398

11192267861882715251296

828385045075911356713

11381218292309596121

212765625560924488938052986593169876

265356025031445215039033811

265356025031445215039033811

265356025031445215039033811

713727162867994013566735

58511582604744880988

8257156235219426106

650203975431184615647

650203975431184615647

182483772946442359576443362853143258

27365188250313503460

27365188250313503460

182193735146243356956410262336142781

161203086839119306845624551524123180

18925956627345096942984416314

199213757984710427125161775415068

837605041254381548879466336

837605041254381548879466336

1111754955645920686495708340

1111754955645920686495708340

159365520103059325751821778

159365520103059325751821778

9333190316311495162

9333190316311495162

37353223287311400271

8328261371229622894104947

6621851063179417783053658

66262954955031042250

5098551433955642261512877147450710

5098551433955642261512877147450710

41491293288388520357

41491293288388520357

8811087118339671441734

41560301406469709374

1171075928990124714531351

1171075928990124714531351

230349220862710301046732439

46561348393462692445

44649399479531841590

60110569891910511474762

579384927117571160547

1201029979940114712811098

59796648696811948716

61233331244336333382

457457834743678454062314461

364356427062881347551313575

819637067379471019778

160166314541286159419561702

78145010571139129017211090

114133010541147142617251400

101810180667580018984128558716

68318363232351358504

579564438284508498569975153

85110065785810131457769

95734501595682975615

667644951075114641788616287

57468275330392614277

369245006236841363350

116103870478810001408787

286218019591795245028382832

123146614151273154621041636

56116583059975611991052

4810477135346691076911

861266773898103716601044

861266773898103716601044

135249017281951234135351835

99156912361256160424201425

349044776957341113384

284318019032541281942252308

8610616528008951494637

11332197249237474161

11332197249237474161

34443292350490565468

18389239321373495260

218185719131529208724182629

876678855368517631217

51422342357408521449

797636826338191133953

15560281377416939344

15560281377416939344

358297824272211292236363337

12510681039893116813581345

82126364288110141470715

30363255291376527388

200146310151080134718371572

12810316347568551376940

373500927533408393362833672

373500927533408393362833672

77595642535803706944

66586611489765694886

28450298331400514299

28450298331400514299

27743246743168996176545207638310693204586

10323158249248347169

295595033504739493175963449

295595033504739493175963449

295595033504739493175963449

295595033504739493175963449

1561167681084712893148312178712868

140814201941911052127101869811330

261251014931756205233862080

261251014931756205233862080

539645743245177570985594701

539645743245177570985594701

586491734193889468564084398

586491734193889468564084398

143256214111829210330841526

143256214111829210330841526

811700957135515152030885

21075163929118195113868138381204349147993

5651432553245131102372255497641534

207227219041651194632562262

207227219041651194632562262

647594944454307509677665546

322301222362017236039002836

312292021282249263838492518

431330728292373291241963639

949157746618341199896

89791461497526935669

727944635456141223657

727944635456141223657

546355126962811324943323421

546355126962811324943323421

352305718852166243935412208

352305718852166243935412208

1042638950334419530577846346

1042638950334419530577846346

241270119862137267733262247

241270119862137267733262247

12168731659265037720112638680

275205813911524175227471957

80522418370575524536

179103778094810681429943

271196814331481174926242037

141192812481242137822211360

435353922972155270040972996

435353922972155270040972996

8669238601966048111115377555

399513031433678428763583610

399513031433678428763583610

302284216961908233833912134

302284216961908233833912134

814456345087736331008

814456345087736331008

717554524325911048638

717554524325911048638

382501529113763424858363109

113175411121327152720051063

1121788913123814171936989

8774633164677642709537027511858504

15711176397299470110381456812227

675444743313902483254215726

863700151505360597587936229

4630339942370820987271473849128144

192802624365696657693

116688528338458765732

196183711231119128522131144

1857378305097588081163

209206313161259150326121522

92420344400451563348

232290512201131156730431434

1466997233636658091081

15712829319329941473925

65531360341463702450

1511648780103111062062798

44365421344479438412

24517121274973132620441667

219148687273593014821079

37366334258379344302

182927746530668962923

420286723222141271534762501

145108076373595912491085

75473466317467427723

311220816151526187124231718

18531201493198362104901509212428

463293023112039260634722930

611370326612560310247033678

298286422242197271035732508

36416201417918124122322544

265280519032103262533622281

265280519032103262533622281

3009245551845217997209873549522509

13251207291909007102581534010805

11310316537858491240642

43585348349374910459

410387435712884325048534348

212333320482642282441542070

9480365171288810691009

9480365171288810691009

85428439340443476747

85428439340443476747

13749924710868718236168188793

13749924710868718236168188793

530346226662449287241443285

530346226662449287241443285

509315421991991242039702963

509315421991991242039702963

369228316921859214428002137

369228316921859214428002137

152250415101997228533881986

152250415101997228533881986

578935496528261244640

578935496528261244640

9015839261317140320711192

348354577046671159453

21559279459468696276

346715838975667544589553359

346715838975667544589553359

346715838975667544589553359

346715838975667544589553359

299603931044404477777262921

299603931044404477777262921

225480324433516362061682165

225480324433516362061682165

68108258976110021317713

68108258976110021317713

326649136335033529784693331

326649136335033529784693331

190409822783343343550901995

41546375438526604327

7222241062173115992591762

131238813121661183733641281

131238813121661183733641281

243295715982088232936311908

243295715982088232936311908

2713276161780520331230173534120603

2713276161780520331230173534120603

6679921570874327984128705840

6679921570874327984128705840

840694547015033591485315765

485415027753127355952473362

345275719021875233432162377

597501535143813425364463985

597501535143813425364463985

264220915281646200527501860

264220915281646200527501860

271256717431717211835972356

271256717431717211835972356

155850728394664903416143636274797082677835

5246257702231113321192863055826187

5246257702231113321192863055826187

26861259811223643593821476713044

676325826341421211237363207

1023443643952607380651314999

671339427751529230941273342

707349927831852272742783694

707349927831852272742783694

445354824951776264039032703

445354824951776264039032703

1218509147312761374662565433

1218509147312761374662565433

4811380022468517111280412874721954

11714351638995177116271936

47491655419645724844

28363453277416541567

60623823461904642965

58549772443874537897

6095305893396621073321583762839175

5533277522968018728282173363734193

488232726241618241527622978

488232726241618241527622978

1820889810079605496081133611943

422203125921537239024252878

626306736442224346940974374

582282128851692282335523573

1758780979495108733491578770

829442839982681391051324511

895325638412364331439034124

1152722665064689672085797539

567318129531783287338053895

553387733122758362245253304

109593125063110226611506

109593125063110226611506

492224331641727289628673617

492224331641727289628673617

492224331641727289628673617

1980088770842124953771968125580111262

279814855144459144140201899517934

82772684424766820734

82772684424766820734

1009389240172589366245644672

1661183880848104714551043

108193421264375225456

23315601153845114922281584

23315601153845114922281584

438284231731784319735834098

438284231731784319735834098

706340131281985291745723917

706340131281985291745723917

253151816231189181419131942

253151816231189181419131942

168597328169062400315733710558992287

221898218993574678481245811220

221898218993574678481245811220

6749230932512212868195273192232504

23397065859542956326980510412

400414407149287714118602006620082

3433200651609610419141413110423431

183599528238520769141507311694

149595887497504169911507511120

392017485164809478138742573321858

749385334401958278258034739

2015746181494564663798759904

1114593347402860430695726881

20415105947115606507678804210938196302

14516755339489043066748997806578834

557315031671406273938943221

557315031671406273938943221

21501046711595630699781066110133

609442936762676384245302968

1508589476893529594259786896

937446457742540449548795127

937446457742540449548795127

16178186112324821864081389035

181931123962712517771233

1130529278843115541954765714

331163228151368250316262654

2129211879886157210611767

111688761424807533738

14615572111772963603660807245

14615572111772963603660807245

627753552523792639358224764

627753552523792639358224764

375191021431111185819941967

375191021431111185819941967

719406650242462415143714461

359199824671070182621702138

276147919071098182415111710

295813003156537290119061450113247

2233961911878563990251065910145

702324536571616280036932954

200510955137235899105181099610854

1352628088543251616464826945

644464448252630431344963870

583929698203557466127223059217053

72121841377541607111210154222

72121841377541607111210154222

503766531544827014432804411402

503766531544827014432804411402

597931041546109911521355

597931041546109911521355

7511737058018741616805

7511737058018741616805

7511737058018741616805

7511737058018741616805

274177219561294214221462441

274177219561294214221462441

914336963209511610295337143961

914336963209511610295337143961

914336963209511610295337143961

914336963209511610295337143961

1015167354197279301111

1015167354197279301111

91540361576339805233334345491378584332177

1960123338785680510393119969083

926576839112934476755153966

900532342073119463152034497

900532342073119463152034497

631395327302232322642402503

631395327302232322642402503

631395327302232322642402503

5972250532215214662211612952525153

5972250532215214662211612952525153

1079367135442190319244174070

1373739755364093558285786337

651250222991569218331702587

1105476136852477363454084215

1017410645672874440244925030

17884980847018851036802408952070793

6186309132329616376251892902523755

343316880126349020137621594412903

834470733982316374345453462

1547714156613952594465055839

3654223551537911281180891965915733

1184633645383268510756424596

828530634352605419747413503

707444831592194356837673268

810555036802824459348723843

6824377752592919529305643427425585

1926999170035411825494057206

763464430562196375940702936

2491137689688733611427123649628

959558436452657426750773422

3674236391810713139199142824419803

629423234852473387345783204

629423234852473387345783204

21111290798957037104411593810948

908543540372678407364424398

462274121921451249030762435

688419233332546343156003810

908612344043473536068984934

908612344043473536068984934

929584941763004461163924655

647367424931910276840462672

647367424931910276840462672

27219331350894149921031629

27219331350894149921031629

4704209291865811989180822335719690

1309646851953699532274275635

1309646851953699532274275635

1451653458073490567976296605

1451653458073490567976296605

1792732669314425645576666691

1792732669314425645576666691

23098342848042150626691883962054602

23098342848042150626691883962054602

23098342848042150626691883962054602

11917465843719529081420714533340752

10304386043094024181350293743534541

2013813465335068716977896866

2014822357574774692279156608

2220789670734991765975497838

2065723861604613686667496865

1542766360174712676375985948

1542766360174712676375985948

84441585373536551739

84441585373536551739

16483681935983738802579637678066960

7121272432550716667240183164528553

7121272432550716667240183164528553

6455271762323014718222442990425852

928423933182263341946033490

994390134352060328343403875

888379728661991296740353153

920412933112283332044413544

752304126591583248635533098

810329430601818273634663445

1060538938792506396361244767

590278920811270204832792656

437248117041163181227382018

718339726901934299136672818

718339726901934299136672818

5479750404598559736639719534144861

167411727895110033117991460710424

1278485857758931075609

1278485857758931075609

1278485857758931075609

527455135644101479555383966

354304626412890341337412907

96796635780840903613

1226138686519607841079

53739439650718922476

1431352805113412701602906

1431352805113412701602906

266194613851604179626861780

266194613851604179626861780

266194613851604179626861780

2511093923875127412631204

2511093923875127412631204

2511093923875127412631204

451266920182132239832912417

451266920182132239832912417

121588468454569695650

13210286898918981302648

38294218287321348278

10651473791321136212592186468848

302657335415030500480913057

302657335415030500480913057

174261116872092216032951585

128396218542938284447961472

7527911544461387358101875682

7527911544461387358101875682

8010966857749691336704

611377986113214051996950

569964747207281190447

989576397279261287711

1061265786103511881595745

54584394445517786416

113746509504594921482

110477640455656638825

1615265091562921458224013396914480

1441249591397019973205863184912542

1441249551396219961205733184612531

3541444466611150310632185474531

1311109873924102514981026

193145910251137144518611195

229219114441702207026901664

18276270294333391319

172185011561430156321341153

153197112111609178626791070

139125210551136145414811140

129105588589510611539983

129105588589510611539983

129105588589510611539983

36462550462547534702

36462550462547534702

36462550462547534702

555687145485258572189664933

330458429433393365561193110

126187510561457146924681082

126187510561457146924681082

193263918391915215335471945

193263918391915215335471945

225228716051865206628471823

32334272248297353273

32334272248297353273

112112074997710401376840

112112074997710401376840

27318252318337353256

27318252318337353256

51503325317385739424

51503325317385739424

32010341493980287527128743462

32010341493980287527128743462

32010341493980287527128743462

32010341493980287527128743462

27423347339403563333

24387330337398500286

24387330337398500286

19375269308318482210

858016705568261027946

49618385390580789534

12374162254302444152

12374162254302444152

40431242303345598247

40431242303345598247

40431242303345598247

40431242303345598247

75183340167370348712

75183340167370348712

75183340167370348712

75183340167370348712

75183340167370348712

75183340167370348712

333270318051581225234502515

59273192110158377399

59273192110158377399

59273192110158377399

59273192110158377399

59273192110158377399

248216815181416193227011777

248216815181416193227011777

248216815181416193227011777

66651467431544943587

31484240331389631204

867976305688001030649

62706491508676965454

357301025324553545733863045

164111410532488300111841413

164111410532488300111841413

164111410532488300111841413

164111410532488300111841413

164111410532488300111841413

192189214652016241422021626

192189214652016241422021626

192189214652016241422021626

38440376508541603402

31399350470482507330

80103362597011171094626

80103362597011171094626

25294234279365365259

25294234279365365259

61655379385544795564

61655379385544795564

61655379385544795564

61655379385544795564

38385256272378533395

38385256272378533395

703490038804094530162944921

703490038804094530162944921

459390527892945363050093302

701408891105011591770619

701408891105011591770619

701408891105011591770619

389249618981895247132392683

389249618981895247132392683

35467244401380501230

61266193135212332326

92549444377520762579

48423278352477596408

24397310791129164412511605

24397310791129164412511605

86625506577755779728

31290175272342321321

58149177155275234278

58149177155275234278

80153313253423202503

9566574694671142942541126946054308

9566574694671142942541126946054308

58560293342418823349

58560293342418823349

58560293342418823349

58560293342418823349

179199014991660209025111566

179199014991660209025111566

178199014991660208725101564

25653329480538878230

17357192278336443188

1428987277628281152873

1428987277628281152873

29226198258224317158

29226198258224317158

113672529504604835715

113672529504604835715

145307478204318337549

145307478204318337549

145307478204318337549

145307478204318337549

256278021162396265135682255

256278021162396265135682255

19532273397397586169

19532273397397586169

21262160257259371134

21262160257259371134

37371247270283534247

37371247270283534247

40309203255267370215

40309203255267370215

849315357477921244613

51680355550546830321

8707504604127737177473316044548376

1305649356344528610176746478

74337308218300383368

74337308218300383368

1209611452344280573972215986

7010525584183193364

592071914790263281

57435302298364474229

91238186159242323392

66290225118215296246

60626411485499710314

84234266165240325398

85792550496661893553

70498383453426686440

90372316188385364378

50378255268363448251

122413411138249662685

122413411138249662685

122413411138249662685

431437928453405401956543001

839255708018641070499

839255708018641070499

44477347382456629275

44477347382456629275

30297373308461430421

22279253247339389275

265260415221891220934221775

768244326056601147438

57471306387472601373

177103782787210291262936

49311283302327464316

49311283302327464316

123725527562690798591

68440311411469491298

629315117016681094356

629315117016681094356

629315117016681094356

6013615021021898156

78595415345455656486

78595415345455656486

78595415345455656486

116804614697756978750

116804614697756978750

116804614697756978750

105490411407505618536

105490411407505618536

54779589733773885456

54779589733773885456

54779589733773885456

43340332350391463342

43340332350391463342

43340332350391463342

174130189687410041453959

93760557521596977638

93760557521596977638

80540339353408472321

80540339353408472321

75582406491518733415

75582406491518733415

75582406491518733415

643348728672822349040113080

62264305275388250322

1208135456367011031628

396200816111556193721751710

77488396463557530365

84455376327452388360

11223119984134242289

112539454399493549528

48383361331429543367

48383361331429543367

355252217791681220828952457

355252217791681220828952457

22351220275268417155

12390186270295528143

98658420396538776506

98658420396538776506

98658420396538776506

715304927132169277038703315

62425333310350599375

62425333310350599375

2019987507048741342922

1027794995736651060521

88195218100159280329

84373241301356494376

84373241301356494376

107264311117203262362

107264311117203262362

105254352146272273517

105254352146272273517

92355356281335510376

92355356281335510376

1661382932889113314521000

1661382932889113314521000

59520421440513683422

57592319324405538299

1085630053324606605674246653

1060619851874562597772636404

1079306556787601258683

91662488582567865437

77380284256419410305

68434291290429366460

81163262148277242478

58617532467578731506

97607359321409634494

62533403287405658432

24249268287309343298

318953107469395112761542

103139328148218170510

103139328148218170510

101484456335369740651

101484456335369740651

88229192146274264306

88229192146274264306

292320022402371289340401874

205242216971788223529261194

308404995886581161334

63632533461752710227

46506332438450549350

877785435836581114680

877785435836581114680

15310336717488491315721

76692445513586926470

76692445513586926470

54512434394549610391

54512434394549610391

54512434394549610391

693310629082037288636793785

35012951484761122113632105

79286310138200389519

58239224124227314268

65276300110190271433

65254286197219212324

64172248113239110418

339180514211272165423091667

113683585501678915709

104373266170276431349

118747561599691962595

388244416761605191725371901

388244416761605191725371901

1098305275716711005550

81519337310410578436

41450271347339448241

103244319174226193443

20016649931000138618511554

20016649931000138618511554

20016649931000138618511554

20016649931000138618511554

104696503499691774723

44510298362453535291

28404228306320567232

20350213290289506207

637619945434918624879876415

74581411435579826696

31408186306329517273

14361156283244481154

1031322989111112801562966

1031322989111112801562966

1031322989111112801562966

66654605601724804691

32432278328355488203

380409627933119375853643523

200297918392323260037951780

23413267366370514223

19303179237258357189

14292162234242424122

459235327087451198497

30394289356389553257

100588396355473739668

100588396355473739668

6309150263215470116

6309150263215470116

1071186601081843827941675531581

192159010221243137121061123

192159010221243137121061123

49389211279317503233

49389211279317503233

708555657327911145517

708555657327911145517

33390232336314630241

37464333396477514275

94171376209337160556

94171376209337160556

190439553399611423844

190439553399611423844

107356481376573369644

697554223354167

15812159831032136415451109

15812159831032136415451109

103111975187310781364728

103111975187310781364728

100275025773813334089221927735

100275025773813334089221927735

99884879761612323906203527558

2581322105311654763

2581322105311654763

1385991286432324

803149173220199

582842113212125

1094882265513343

1094882265513343

181447309297430431537

181447309297430431537

771878116120203

246100167208626697

246100167208626697

8508374362256592636131723058

8508374362256592636131723058

68514863912138

132791048424725197571135812458

112473635490738605945

112473635490738605945

79390508384534541709

629501739173991521962404780

54303289249344334401

564470635773724483759044308

10712919391010128115621010

29366217253285516306

29366217253285516306

54838544653822983462

54838544653822983462

84464393452599559535

84464393452599559535

84464393452599559535

110117987893610551498912

105114381389910001436849

105114381389910001436849

124798590541799855821

51486351349479558462

569351937372666365744166551

476311332102413314839165708

111120284280197514321021

43453317284319450388

67726520516647974618

22493276338350777242

71273302281403336382

69254274270372324326

69254274270372324326

1032185260132342751

98107201424102582

162143310771177150119141363

98112081292711341578956

11512259408400820198

92406513253507499796

83342294163315401535

83342294163315401535

83342294163315401535

83342294163315401535

83342294163315401535

83342294163315401535

100517477407675585724

66365244277384408345

59267186206289268263

59267186206289268263

59267186206289268263
