# Supplementary material for: Relative abundance of ‘Candidatus Tenderia electrophaga’ is linked to cathodic current in an aerobic biocathode community
Source: Microb Biotechnol. 2017 Jul 11;11(1):98–111. doi: 10.1111/1751-7915.12757 (PMC5743799; doi:10.1111/1751-7915.12757)
Supplement: Supplementary file 5 [file MBT2-11-98-s005.html]

Javascript must be enabled to view this page.

richness


1031813
2021213
2031813
3040813
4040813
4021213
4032113

20172871411159151728390812401510178972222768333221211929

1072624720745279268999174608251

1072624720745279268999174608251

1072624720745279268999174608251

1072624720745279268999174608251

9564521989373587590937638815017395765

1989398115882083185930411501

1690353713781663156625431318

1690353713781663156625431318

47431271936714326513677495308

47431271936714326513677495308

47431271936714326513677495308

3625706035802812455954504754

1845352716841276209425992515

7955218451559842463445938512566076873

19254473981581916813199253302320462

46221290534874295474387174542

2941634623102866259544383134

3089672829152637353553413785

1535409414541345169924821436

1337403611621353142824141250

585411321874263227651374778985554975

1640323315851178188728731598

40761928492740612089187496185337264

103382494189291052311405165989950

1321586269190911701111020903131430219721131561174

2022405415451584194527611806

2022405415451584194527611806

1819365713281472169025571413

1800363312971465161724581261

1997536121521788283934742692

1997536121521788283934742692

1997536121521788283934742692

1997536121521788283934742692

3132692229692339324548253950

2354581524021654220833873260

17945919371653961545883910840874158223542441579418518581

65707451354954174583404643068784087980766977387782

329054642733430562216264322682696333633451

42765896325143432171459208440072421

176320882039938132220042736

176320882039938132220042736

13635283351676910763144872698122824

13635283351676910763144872698122824

536712065601937286076109369079

536712065601937286076109369079

2459643222242055222748172816

2459643222242055222748172816

50301021767203977571997589826

50301021767203977571997589826

48191042351413483546498226874

48191042351413483546498226874

233939446790300016148610223493490241444383

2170728934361139184162942687547903

2170728934361139184162942687547903

5776149256175385457311623011566

5776149256175385457311623011566

21783419062925616860240004469940327

21783419062925616860240004469940327

23358438152750316934255234632439990

23358438152750316934255234632439990

39770850625034828283403139454475908

39770850625034828283403139454475908

28599477813330017064265464588744209

28599477813330017064265464588744209

22043521252552415475228786336544616

22043521252552415475228786336544616

46325744795450725357375297891076741

46325744795450725357375297891076741

30521632533478420989315864959540259

73654151507847366234490674115660108925

7709126209380514088901024113229

3611557845732255401945886195

3611557845732255401945886195

4022674346622772470453906722

4022674346622772470453906722

6362413526872304551227805510193491587

685012772771858158469998011494

685012772771858158469998011494

517611854702939456818100919248

517611854702939456818100919248

56591056767904721624491648194

56591056767904721624491648194

14239395491328814551170782440213957

14239395491328814551170782440213957

4040883149133886537268246509

4040883149133886537268246509

81791599595566761105091268911876

81791599595566761105091268911876

7211126987885589087061066310916

7211126987885589087061066310916

105043313382201105567768471110531312055561041425

9049148999299566789381154710555

3637521936661960319341073996

3637521936661960319341073996

4644882848533422521967355830

4644882848533422521967355830

974524851109919060132952171313656

551913820593451327063122007006

551913820593451327063122007006

38391011146683554571186556149

38391011146683554571186556149

148401185491190889121037161947175041217089

20562348392603426297342403660732755

20562348392603426297342403660732755

62726509238444625406350673856187652

62726509238444625406350673856187652

2404472225481941294632602912

2404472225481941294632602912

10848153361305110110136901448113368

10848153361305110110136901448113368

15259255931958519825269722913125200

15259255931958519825269722913125200

1089521978124987832113931891616116

1089521978124987832113931891616116

527659374673485387332972478951343199317610

527659374673485387332972478951343199317610

527659374673485387332972478951343199317610

18660351892144314321218482973625621

1276123067144799639144231985717507

1276123067144799639144231985717507

53981115464394327688091807606

53981115464394327688091807606

13220287311701811151162502736021757

471751055125341537174539079617067686

464710515534239265429105847079

464710515534239265429105847079

1046320464107707686108051910314111

1046320464107707686108051910314111

3122832334843367415672203866

3122832334843367415672203866

475312290561841096290109727318

475312290561841096290109727318

1274026806151249668142482347619117

1274026806151249668142482347619117

35376623894289524446373855546451620

4072776651582763427879656250

4072776651582763427879656250

15127277981863511601172922625523964

15127277981863511601172922625523964

1478924537174779028144791950819534

1478924537174779028144791950819534

17087443361958414332223114050422096

39189886510634355701109166198

39189886510634355701109166198

490114047559839296466135056167

490114047559839296466135056167

810219935873868209992154249516

810219935873868209992154249516

532161122346053641898615509947275850

16073312581587911725166322608620119

16073312581587911725166322608620119

1172826347150689704141432310719040

1172826347150689704141432310719040

984421693105348010113482002614001

984421693105348010113482002614001

26816578873161221496325035056242068

43201079249593539559587076647

43201079249593539559587076647

13000270661594310908159192403621644

13000270661594310908159192403621644

922219231102116751105661692213103

922219231102116751105661692213103

6626174627734621491871628410831

2585673428532236372654473504

2585673428532236372654473504

397910584483139105424107577268

397910584483139105424107577268

485221082275764838040578438750772914

485221082275764838040578438750772914

19581398402269015363220393199628493

19581398402269015363220393199628493

16847367682045013095202782976825892

16847367682045013095202782976825892

43251135353213839569583246327

43251135353213839569583246327

571414441635241877082125638679

571414441635241877082125638679

3757738848862764429177206951

7726120359510504488301060512744

7726120359510504488301060512744

7726120359510504488301060512744

7726120359510504488301060512744

869744173036122720169965912518052637991441990

869744173036122720169965912518052637991441990

2371435528531541255539834638

1492267217591000158423462463

52567619671008574171074833511027

52567619671008574171074833511027

52567619671008574171074833511027

52567619671008574171074833511027

4306760832522516354249494511

4306760832522516354249494511

4306760832522516354249494511

4306760832522516354249494511

2986576881142531025031991591359969237435072320891

11931682669317581792089173170508383140515

1474526292182617193127982031519788

1474526292182617193127982031519788

74077261041168209667145282199072566

74077261041168209667145282199072566

79671690629431313605568410588311804

79671690629431313605568410588311804

2220542631201652376439524166

2220542631201652376439524166

988245524312593273415801727711717898

988245524312593273415801727711717898

1148748315841550301447711990

1148748315841550301447711990

2833071789243228662951876930338443331749182129351

72391230994164204737798809556

72391230994164204737798809556

4612962963073275539774705605

4612962963073275539774705605

75711175091134019630290419474

75711175091134019630290419474

48851016082214017651082238671

48851016082214017651082238671

62491286210868454478431001010403

62491286210868454478431001010403

3090744839142433383658474846

3090744839142433383658474846

85411620016264472685971149511788

85411620016264472685971149511788

1370933052155559398163181772413390

1370933052155559398163181772413390

3485775044022816421059994652

3485775044022816421059994652

4220751170202542407968076747

4220751170202542407968076747

65101384323626418795701176113624

65101384323626418795701176113624

1456820917750517959230571375531172

1456820917750517959230571375531172

819115113147026664100321253313869

819115113147026664100321253313869

982221293134317022108871566013696

982221293134317022108871566013696

1524318524091337188018502338

1524318524091337188018502338

3573618441951862380748155443

3573618441951862380748155443

3582718439972008351753054509

3582718439972008351753054509

1011419031138097150107741615416152

1011419031138097150107741615416152

74181601410897494482291294110682

74181601410897494482291294110682

64241246810575513078831053310306

64241246810575513078831053310306

52501070063683651616374526396

52501070063683651616374526396

104622236715523671898921841715472

104622236715523671898921841715472

1010116393204536807119701223917066

1010116393204536807119701223917066

2733418938601545300135124670

2733418938601545300135124670

5085952448833119556466796492

5085952448833119556466796492

14705312982235310323165372092119530

14705312982235310323165372092119530

1063720987125468870129912099116932

1063720987125468870129912099116932

2533864729981522857661680562302545627582371679374

2533864729981522857661680562302545627582371679374

3238602737612851360857184210

3238602737612851360857184210

191871415530163911173246216200597351226290

14972401461221212141137922678912760

3085774224822617338565123305

3085774224822617338565123305

3085774224822617338565123305

914624117728673837388150266967

914624117728673837388150266967

914624117728673837388150266967

2608774323351989281849732408

2608774323351989281849732408

2608774323351989281849732408

744715719612370668156122098048

2015418716762046219829652311

2015418716762046219829652311

2015418716762046219829652311

54191153244235018593591945728

2083355813421346150931272096

2083355813421346150931272096

2511584123462697319945382808

2511584123462697319945382808

121637811749950171153194316

489561009984686647522602036699763175

71771385764976053812488178276

71771385764976053812488178276

71771385764976053812488178276

25582503622485725314316833453534742

9954187641027010197134151425514957

9954187641027010197134151425514957

14995311341416814727177861982319399

14995311341416814727177861982319399

14569336101413313981180642166118248

90252039584667348104941281111415

90252039584667348104941281111415

54081301155356526738488156773

54081301155356526738488156773

99062796780841041311043177108082

99062796780841041311043177108082

99062796780841041311043177108082

99062796780841041311043177108082

16354337071426010961154922456620859

5485969444493451438965665413

3198510922251520222635173168

3198510922251520222635173168

2287458522221931216330492242

2287458522221931216330492242

54901228944443109431985546269

54901228944443109431985546269

54901228944443109431985546269

53571133453214377660390909035

2052500622392229359736024523

2052500622392229359736024523

3283604830292139293753744413

3283604830292139293753744413

6130311581050835524657332038892882348

15745389081225312750148192585212278

1290133772101851142813141217729463

1290133772101851142813141217729463

451327624238331394725824136259269559

227832860921603235273977032898949361

227832860921603235273977032898949361

814420245615565077091138296714

814420245615565077091138296714

2942515321241808239144593020

2942515321241808239144593020

55561064145833363428479255930

55561064145833363428479255930

3388701022452222252741092402

3388701022452222252741092402

32389903672555434803362516251824023

1239134351102061282613743236019279

1239134351102061282613743236019279

1239134351102061282613743236019279

1239134351102061282613743236019279

3892948634022800360570883806

3087749227172315281250122667

1425386011681337166224681103

68052511345665143513922881117387094990063286376058

43811459835374972515199554090

43811459835374972515199554090

177943165611009442380382190317956

9237319774363377476765888313279183503

9223619759763226475415867213246383315

57051255539892399373676865149

57051255539892399373676865149

27457667742057220327220124237524882

27457667742057220327220124237524882

570971120873738623423314587863551679

570971120873738623423314587863551679

3182774031823158392451013330

3182774031823158392451013330

3142754630693026381749943141

3142754630693026381749943141

96755245325774669404510193616041785072

96755245325774669404510193616041785072

26120658382076026289291524638922894

26120658382076026289291524638922894

17351310521315610328112551884616741

17351310521315610328112551884616741

16469447081342017609193113065514032

16469447081342017609193113065514032

31511879202520633690351685382326607

31511879202520633690351685382326607

2728738424352633366653363125

2145529516891785264441032047

1111277188894414132812942

1111277188894414132812942

20921974116884128122928529162255726925931916922

2966512820211149174429613056

2970314220591211152416692753

2970314220591211152416692753

2970314220591211152416692753

25808503741949014984186923595827064

25808503741949014984186923595827064

25808503741949014984186923595827064

620818633437728434240108706246

620818633437728434240108706246

620818633437728434240108706246

11571205277956284746571239010787

11571205277956284746571239010787

11571205277956284746571239010787

2211334614451272187331401840

2211334614451272187331401840

2211334614451272187331401840

13251266648967571789642113711999

10839211007083390870951619510099

10839211007083390870951619510099

20005203934415121696924870457041025716961829149

4285787929382325271454034056

4285787929382325271454034056

27738586491832310605164873873630371

27738586491832310605164873873630371

17766783505780107087917847846584122854271614095

17766783505780107087917847846584122854271614095

9963917602465346343414646611918489761

9963917602465346343414646611918489761

144038381219986140130981319

144038381219986140130981319

12038252238456506768711805312155

12038252238456506768711805312155

12170216038370497367391364711444

12170216038370497367391364711444

46641063636771653288075955475

46641063636771653288075955475

31111563924612276330378983402

31111563924612276330378983402

37594831002601725257310745747333902

196542438074153095156645184364294594190289

20712480791604516054192583302721689

20712480791604516054192583302721689

20712480791604516054192583302721689

174085385257135484138843163460258698166979

20111470241539516285190143046618552

20111470241539516285190143046618552

18975365361452114607169142699218144

18975365361452114607169142699218144

1188431830975210587120951966611565

1188431830975210587120951966611565

28921545582110715641183623321727327

28921545582110715641183623321727327

16376371781319614467166102491015235

16376371781319614467166102491015235

927324478758077389757160809685

927324478758077389757160809685

18368406971514316245191812945817690

18368406971514316245191812945817690

119072243899872946338186108167872116520

119072243899872946338186108167872116520

2957779526332048268457153310

2957779526332048268457153310

4021860327792644360067773920

4021860327792644360067773920

2922769724371745258449742896

2922769724371745258449742896

6758640345931580239749136672

6758640345931580239749136672

3391433321241206189832783646

3391433321241206189832783646

864018390657658927077135738098

864018390657658927077135738098

556215056454147235442113435018

556215056454147235442113435018

55031138843243084451284265658

55031138843243084451284265658

3676703325631405247651293332

3676703325631405247651293332

4899919032893057409861354900

4899919032893057409861354900

3375707919941469193335702964

3375707919941469193335702964

2384533313411276195037741889

2384533313411276195037741889

251732752256768110325032726

251732752256768110325032726

235835891761941148826892363

235835891761941148826892363

61341269344533736488188106180

61341269344533736488188106180

14854897101064193522951302

14854897101064193522951302

2857517118261658192439172944

2857517118261658192439172944

617021458574455926393391818475263049

59591160645272659369880935818

44921067334142694298782094250

510241216603619420848322156763452497

35568864772427312401194084357734487

35568864772427312401194084357734487

817372779491396317202801368170648327955223

140880231680871722482043016157059130754

131673215293811022187938390143759121767

131673215293811022187938390143759121767

883915227580828884449124338742

883915227580828884449124338742

730318803561434364456128256371

1020622589761352007176158729892

15020232058119404154101337217205

8247116276852446257304029111246680097

518611035453377520028296517330752100

518611035453377520028296517330752100

269885299416363529095163545724794

269885299416363529095163545724794

873419628581126024312142478648

530924268927218301131411254015298912677499

520307256139211777128613249256289686665817

520307256139211777128613249256289686665817

980312714628627574629869411108

980312714628627574629869411108

20571941739513457298192129371281106197091

20251741022913202796903127485276059193656

674471478834551236410467659481063407

674471478834551236410467659481063407

826519906578346765915121967743

826519906578346765915121967743

106953203488688094562062465143082103948

106953203488688094562062465143082103948

3132694222891254183248503228

203863966414672623698922621219950

178483660712897564990822365917642

643917747482119493955111616695

643917747482119493955111616695

2322481013381093142330272043

2322481013381093142330272043

5630720740311352206954865515

5630720740311352206954865515

175841211394750109523051541

175841211394750109523051541

54269844763467221790323085831655226

54269844763467221790323085831655226

54269844763467221790323085831655226

54269844763467221790323085831655226

1028823025597925011808511745619816565256

1978447613401691192639021482

26297548701854214359189763906525159

35003554352550816695214673766638061

2023511714851217174229662109

32819496042390015251195913409235716

829191696013059782555685

829191696013059782555685

4378850626862628374262864148

4378850626862628374262864148

3064847122212794313453302473

3064847122212794313453302473

2304527497164717267104061802626354

2304527497164717267104061802626354

72024419453165635807097947494391267508565713

308795770104232527263572285242506641255056

35129738642410927086269025088226659

35129738642410927086269025088226659

7645619681558237666107240712934462469

7645619681558237666107240712934462469

23161682191953425806273264563418603

23161682191953425806273264563418603

28003739182210226719281545067922703

28003739182210226719281545067922703

473391288103569146427462818055037075

473391288103569146427462818055037075

13566285798566739993111994112490

13566285798566739993111994112490

547811215604130337444442307955151875

547811215604130337444442307955151875

338658960906273620365968384454623436261093

30085776402308425880290905151224351

30085776402308425880290905151224351

24937752862198828990294015081921068

24937752862198828990294015081921068

29719883642402134594366075934323006

29719883642402134594366075934323006

396581114343119444505446967211329879

396581114343119444505446967211329879

28946760802325828565293115152922161

28946760802325828565293115152922161

29081771422322831204313175039522134

29081771422322831204313175039522134

30891849342506933271341155450024024

30891849342506933271341155450024024

25121713742115225862286854297719756

25121713742115225862286854297719756

3651732726351917223957733415

3651732726351917223957733415

27458801082264629764319585221121041

27458801082264629764319585221121041

675719994645973207613116956248

675719994645973207613116956248

675719994645973207613116956248

839018077606965487900131537595

839018077606965487900131537595

839018077606965487900131537595

839018077606965487900131537595

10650962597250852924875908102847617403741050865

19595563271581719556223003809519130

19412559061561519499221293790018897

3031784720292116289252012925

3031784720292116289252012925

837625157682989939674164217509

837625157682989939674164217509

680718706544466197759137896322

680718706544466197759137896322

5192431157922424992382234471678802331567429

31717708662447917411237694798236772

26472420155438254414753133

26472420155438254414753133

5060798734312210329660585407

5060798734312210329660585407

3256763527822823319054493973

3256763527822823319054493973

3256914423011766238158713197

3256914423011766238158713197

1205931012103427591103412106715662

1205931012103427591103412106715662

17675471431487315479173622918416402

8585243127537664494731720011512

14161310001147710318113852096514617

12927330861145512264137262119912257

7172116988359205551086750411611282887

16069378551229612989153302666614876

16069378551229612989153302666614876

15842446661436011549142122668924224

15842446661436011549142122668924224

100602381079738263104771742710249

100602381079738263104771742710249

4301936734682804344054064204

4301936734682804344054064204

4110811126512819333464883982

4110811126512819333464883982

29678637822301123235271844243429743

23756492632197618998241973518527807

458191009623703330542399746692349997

51851120345133629537672445837

51851120345133629537672445837

1074024801106617611105271626714017

1074024801106617611105271626714017

5446807842422968365652115904

5446807842422968365652115904

674615925480824714188112336876

674615925480824714188112336876

61341446341164659594890825607

61341446341164659594890825607

981122125744473808393152689786

981122125744473808393152689786

161822340216132870115156141936245704177969

15673280731249210838126372039417715

15673280731249210838126372039417715

594913423468353506423107056382

594913423468353506423107056382

95412222279527476100221414710810

95412222279527476100221414710810

9052178608058568278171430111342

9052178608058568278171430111342

106472948889457911105341924712812

106472948889457911105341924712812

115152422698409558116211790211811

115152422698409558116211790211811

12889279151058810075117492073714947

12889279151058810075117492073714947

10737244179678831595681812113547

10737244179678831595681812113547

665313963518241545240113086658

665313963518241545240113086658

1188025570104998792109871841414179

1188025570104998792109871841414179

77171309554793841457894266944

77171309554793841457894266944

131802875790468922103271937910985

131802875790468922103271937910985

1429927578119018976120801968816076

1429927578119018976120801968816076

142793358352111874120180136132237278129377

142793358352111874120180136132237278129377

43791279835004441502395963920

43791279835004441502395963920

19266538671591616113199293398820632

19266538671591616113199293398820632

16133467521193613425153072929712888

16133467521193613425153072929712888

27611550672091719940224243880325374

27611550672091719940224243880325374

15388341601184110734125672281514587

15388341601184110734125672281514587

31775760432475429566316025328727088

31775760432475429566316025328727088

17051425381294914878160892654914688

17051425381294914878160892654914688

119932959599439926115082060712803

21552581871641623927241994005915147

21552581871641623927241994005915147

20471553811574022909233043802314390

20471553811574022909233043802314390

8430419689065175692428131113120577376

30292670332187123105275234497726762

978822048644962968001145768824

978822048644962968001145768824

20084441271512016636191812977717490

20084441271512016636191812977717490

510531222214088242855497248094647894

20264511681634618775217493422518843

20264511681634618775217493422518843

1942497414901302214234581909

1942497414901302214234581909

18553426731449015317171452887316634

18553426731449015317171452887316634

58821259045343606464677655516

58821259045343606464677655516

10426257907917971510803173749735

10426257907917971510803173749735

43471092334553920460984533808

43471092334553920460984533808

60761479944365720617488925860

60761479944365720617488925860

6844519674954773777087775811984945059

6844519674954773777087775811984945059

35066988692735939776380466081723265

35066988692735939776380466081723265

32588947112653937121383005730521235

32588947112653937121383005730521235

114007201292120063102926156370540412185682

670214715531147236419101537392

670214715531147236419101537392

3159672322112030284145783157

2751622420061910270741212831

3336790429702663351554753932

1543288315281201180923991878

48651284050145416692693775522

48651284050145416692693775522

48651284050145416692693775522

48651284050145416692693775522

958401511011541196627451051

958401511011541196627451051

12641285931162111849151471895113587

12641285931162111849151471895113587

796017487781475849970115708854

796017487781475849970115708854

1579323217731495198924672040

1579323217731495198924672040

2091490416632314257528361552

2091490416632314257528361552

3135684330802540378643143496

3135684330802540378643143496

43681056835824012476669594552

2258544418632474296335842138

2258544418632474296335842138

2258544418632474296335842138

2098512417191538180333752368

2098512417191538180333752368

2098512417191538180333752368

19757351061795417481235082543525065

95781864494349143131331344513616

82741773185788617122391264812117

82741773185788617122391264812117

2102558925132496360440893799

2102558925132496360440893799

1479368815761488224823301774

1479368815761488224823301774

3704584528392701326145783613

3704584528392701326145783613

5859957651555028612964906542

5859957651555028612964906542

3068378623641972221725362218

3520341195771378775035310293884007410662

1032415070699350287230887011803

1032415070699350287230887011803

180425451142561125015442247

5114708632952690348244315472

3176542324071718237228463744

1943382313741191158717202279

1943382313741191158717202279

652415797642179789897110017965

652415797642179789897110017965

2318687626383480426649492902

4201884537414381542660354949

22424314181690610178166342226727088

22424314181690610178166342226727088

4863448225801024243030295916

10400180828979589792451224912734

1984397216341090177823712301

1984397216341090177823712301

6183830147422777420763807259

2929530325071693250340252909

2929530325071693250340252909

45671002338043023488976215912

45671002338043023488976215912

2836964065993782199195520428752330075

2772113946791603192135363727902322155

2737123723090008184405208926383317729

30374219701845403403513

30374219701845403403513

20841385871183481022363

20841385871183481022363

6849396837272637524629948669

6849396837272637524629948669

512245715503239803925863

512245715503239803925863

25769778493837716062845

25769778493837716062845

8395908251576919928409844

8395908251576919928409844

536339415462299153116475

536339415462299153116475

14021712411418404747174769165162137

14021712411418404747174769165162137

14262317801285112029161482201318103

4077964938364276567169955432

4077964938364276567169955432

4077964938364276567169955432

4077964938364276567169955432

4077964938364276567169955432

962209212631256148615791711

45661218743624526593681855625

45661218743624526593681855625

1841553215741800267034452037

1494394315551515166827502115

2932512619561301175828963129

2932512619561301175828963129

2932512619561301175828963129

2932512619561301175828963129

2932512619561301175828963129

50981010942934170465780944209

50981010942934170465780944209

50981010942934170465780944209

4015882633323843403273763506

2445615819921960254642812360

149867300474144398136470175063220417198056

3001918736943192430555244969

3001918736943192430555244969

3001918736943192430555244969

3001918736943192430555244969

3001918736943192430555244969

1100327813561246190626031627

1100327813561246190626031627

1100327813561246190626031627

1100327813561246190626031627

1100327813561246190626031627

2632390723351704197235312879

16907320261709514864186212190322788

4454824739483536409460575125

4454824739483536409460575125

4454824739483536409460575125

4454824739483536409460575125

12433237511312811307145251584117620

63731084159964866658177738475

63731084159964866658177738475

63731084159964866658177738475

57701232268976180760877948759

57701232268976180760877948759

57701232268976180760877948759

120610242607114474110316141242179987157696

118047237750111942107879137806175925154118

1700383616661463180231462354

1700383616661463180231462354

1700383616661463180231462354

17252314261485812189166242278522272

55501105148104280552280127077

55501105148104280552280127077

2161371322881484225526553532

2161371322881484225526553532

2552404724251569233529193333

2552404724251569233529193333

3700688630913203378048514419

3700688630913203378048514419

95861949890939341117071491211601

95861949890939341117071491211601

95861949890939341117071491211601

20891389951891417167226652930125428

9033171127595686889491226010377

9033171127595686889491226010377

3229609229952553369541353768

3229609229952553369541353768

8376152487933753897031214110812

8376152487933753897031214110812

14861309901601915458199422489421906

1823347521522423297531842747

1823347521522423297531842747

1843363223672267291437563256

1843363223672267291437563256

2311426123872149290334233030

2311426123872149290334233030

1623329418001621209129832628

1623329418001621209129832628

1943392625692470337728853326

1943392625692470337728853326

2384460219102249224730802572

2384460219102249224730802572

3739748437873953482554785491

3739748437873953482554785491

3739748437873953482554785491

43481101838604597524877295575

43481101838604597524877295575

43481101838604597524877295575

689114038619662397511104357513

689114038619662397511104357513

689114038619662397511104357513

4194865940353621492963405847

2076392619011633226327302645

2076392619011633226327302645

2118473321321969266636103202

2118473321321969266636103202

2897655928953262346744513862

2897655928953262346744513862

2897655928953262346744513862

15406324171416213100170232290919669

54691198346994858568488076419

54691198346994858568488076419

4586920042743436436762506325

4586920042743436436762506325

4655978543494183589969035936

4655978543494183589969035936

713615954725971088851115469485

713615954725971088851115469485

713615954725971088851115469485

2385471522172159294638113070

26115576233197753963689993874343535

4075943990342573633823672212904

4075943990342573633823672212904

4075943990342573633823672212904

4075943990342573633823672212904

4075943990342573633823672212904

21912478142216425989319883197129317

21749474742195425864317013170328859

21749474742195425864317013170328859

3942732235534571530957684576

3942732235534571530957684576

1508451017901795233530032180

1508451017901795233530032180

1890734220072639267838562247

1890734220072639267838562247

4561704645895368689742016549

4561704645895368689742016549

126829328991353172918921479

126829328991353172918921479

1040282411621366154318251404

1040282411621366154318251404

3313629529962768329344253980

3313629529962768329344253980

2240396823582652379829293381

2240396823582652379829293381

556361116224951041252563568980272080

34398680892837525484348614890437814

31968617672635924259322124463735126

3342627332372758373945503776

3342627332372758373945503776

3342627332372758373945503776

1979531014352047253435351517

12734198667140915662861482

12734198667140915662861482

4310821536932667366661365313

3880675034633137445047223925

3412603929822962410140783386

3412603929822962410140783386

3430636131652953387049184006

3911754232352606409751034783

1319526813139149568130441912723946

1259525348127529200123021756021895

4404976342223540472967605730

1621376216691548175524911966

1621376216691548175524911966

2144344717361943242528143169

1358222911661359147417091859

1358222911661359147417091859

2058434720671395211534392864

1532120971574110

532112124450543545872178026134

532112124450543545872178026134

44759614411736204977163095609

66013037896001328105041183

66013037896001328105041183

1852248515171140141519662345

1852248515171140141519662345

1852248515171140141519662345

15983378771326414357185042548217316

1639493512441338172832442044

1639493512441338172832442044

1639493512441338172832442044

1639493512441338172832442044

1639493512441338172832442044

1650453613001138164628651851

1650453613001138164628651851

1650453613001138164628651851

1650453613001138164628651851

1650453613001138164628651851

1922297315511476197026652273

1922297315511476197026652273

1922297315511476197026652273

1922297315511476197026652273

1922297315511476197026652273

636916122526263278303105956717

48011147139444432571574995219

3904950033403644467160884469

1349384212221592227228091371

1349384212221592227228091371

1979472316031903228130581289

1960472315841891223930411198

41771352339934162506276375775

41771352339934162506276375775

41771352339934162506276375775

41771352339934162506276375775

1326361910781409145819341203

1326361910781409145819341203

1797541617801883201035452003

1797541617801883201035452003
