# Supplementary material for: Relative abundance of ‘Candidatus Tenderia electrophaga’ is linked to cathodic current in an aerobic biocathode community
Source: Microb Biotechnol. 2017 Jul 11;11(1):98–111. doi: 10.1111/1751-7915.12757 (PMC5743799; doi:10.1111/1751-7915.12757)
Supplement: Supplementary file 6 — Fig. S4a‐b (Krona plot, download file before viewing in browser). Interactive Krona plots depicting relative abundance predicted by 16S rRNA gene amplicon sequencing of each hypervariable region for eight replicate bioelectrochemical systems (BES) using OTUs generated by CD‐HIT with RDP classifier (a) or mothur (b). [file MBT2-11-98-s006.html]

Javascript must be enabled to view this page.

richness


V12\_AllSamples
V12\_1031813
V12\_2021213
V12\_2031813
V12\_2040813
V12\_3040813
V12\_4021213
V12\_4032113
V12\_4040813
V3\_AllSamples
V3\_1031813
V3\_2021213
V3\_2031813
V3\_2040813
V3\_3040813
V3\_4021213
V3\_4032113
V3\_4040813
V4\_AllSamples
V4\_1031813
V4\_2021213
V4\_2031813
V4\_2040813
V4\_3040813
V4\_4021213
V4\_4032113
V4\_4040813
V5\_AllSamples
V5\_1031813
V5\_2021213
V5\_2031813
V5\_2040813
V5\_3040813
V5\_4021213
V5\_4032113
V5\_4040813
V6\_AllSamples
V6\_1031813
V6\_2021213
V6\_2031813
V6\_2040813
V6\_3040813
V6\_4021213
V6\_4032113
V6\_4040813
V78\_AllSamples
V78\_1031813
V78\_2021213
V78\_2031813
V78\_2040813
V78\_3040813
V78\_4021213
V78\_4032113
V78\_4040813
V9\_AllSamples
V9\_1031813
V9\_2021213
V9\_2031813
V9\_2040813
V9\_3040813
V9\_4021213
V9\_4032113
V9\_4040813

1202304138809228081495822202743024837164711812917857225484515203555398036032936742644810133995610104435726088699512384568008822501740821239311284319459591853585171610363358261028003048139949916007814084340455856933699643980533887025498305466349203554095938269247105948938715339852252463614787136941289114275134549601811733621120931125649110993458406657444831317996

5050000050500000000000000000000000000000000000000000000000000000000

5050000050500000000000000000000000000000000000000000000000000000000

6407715182963364733410333502121074520000000000157433012743360298128914931162182103764426581183000000000000000000

6407715182963364733410333502121074520000000000157433012743360298128914931162182103764426581183000000000000000000

0000000000000000000000000001574330127433602981289000000000000000000000000000

0000000000000000000000000001574330127433602981289000000000000000000000000000

0000000000000000000000000001574330127433602981289000000000000000000000000000

0000000000000000000000000001574330127433602981289000000000000000000000000000

640771518296336473341033350212107452000000000000000000014931162182103764426581183000000000000000000

640771518296336473341033350212107452000000000000000000014931162182103764426581183000000000000000000

256920322125373115661565001000000000000000000000014931162182103764426581183000000000000000000

256920322125373115661565001000000000000000000000014931162182103764426581183000000000000000000

29915121682952163184431785212974520000000000000000000000000000000000000000000000

29915121682952163184431785212974520000000000000000000000000000000000000000000000

8470000084700000000000000000000000000000000000000000000000000000000

5000000050000000000000000000000000000000000000000000000000000000000

3470000034700000000000000000000000000000000000000000000000000000000

000000000140014000000000000000000000000000000000000000000000000000000

000000000140014000000000000000000000000000000000000000000000000000000

000000000140014000000000000000000000000000000000000000000000000000000

000000000140014000000000000000000000000000000000000000000000000000000

4137139719646345263010594085688320441876851047456973563265141426143802200971748811282801371777291195760011446981201211566108500000000018352434518726811786125511950282951

4137139719646345263010594085688320441876851047456973563265141426143802200971748811282801371777291195760011446981201211566108500000000018352434518726811786125511950282951

4137139719646345263010594085688320441876851047456973563265141426143802200971748811282801371777291195760011446981201211566108500000000018352434518726811786125511950282951

4137139719646345263010594085688320441876851047456973563265141426143802200971748811282801371777291195760011446981201211566108500000000018352434518726811786125511950282951

0000000000000000006326514142614000000000000000000000000000000000000

00000000000000000000000000000000000000000000000000000022576051714935736128821

00000000000000000000000000000000000019891112692294119033163683000000000000000000

393227338711303398000000000000000000000000000000000000000000000000000000

3744137512613258150010263105688320441876851047456973500000000038022009717488112828013717753021846331915287308814034020000000001609542851822510129368211349002130

1156189137363227801488942198313021053007711703917810024911244817755383435846036578744355833974210022134774787533512380967905820341707071185061284189427789679583715210332948240977987988124329883117805164316655653833235093946553858315445875377328896253876137788945509248791715339842250461514676136941288113173249129490631718981100610122374310521508343477224881270613

500084433657001717551083311331523175411599214610642641911191703018331116914915631711314646369187823286561227011398326497847705191921711238814109927503083873043142554335534978614857402854252324332292716603121467091585602974713360835038023275517639926195543772694061705941432130816182301245538615693770503233551538401617314871205183556087

5974454978619920850212954413328867813684120461409423375698074034849714220118112321113811718155529752651775536151561407702525636997160879286373863812825553202343944012553137521233447351388297359727327000000000295896548442002762534146041914110092329345121

5974454978619920850212954413328867813684120461409423375698074034849714220118112321113811718155529752651775536151561407702525636997160879286373863812825553202343944012553137521233447351388297359727327000000000295896548442002762534146041914110092329345121

0000000000000000000000000000000000000000000000000000001523315212669654056381411793863046

00000000000000000000000000000000000000000000000000000010025358236226193327575482653

00000000010618014559383813139417733184718712812715000000000000000000025026282861006728309672347348530000000001353713271700064051927

0000000000000000000000000000000000005624208328841113241401574153200000000012986328017362322459708733130

0000000000000000000000000001478935054200010000000000000000000000000000

438194153811673061397563412318610100000000000000000021760161767141365348969533103373000000000000000000000000000

128986163212418924976090623812040331822461121967365225120242393512230638477812294240641411096032350127324849103337847662593081712628985065271641580000000000000000000

0000000000000000002196733559607593315334000000000000000000000000000000000000

15478067000905765502243836743197471312850000000000000000007108634511128412828606362000000000000000000

0000000000000000001669191714891099120559375003323565000000000000000000000000000000000000

1606551868397131037117000000000000000000000000000000000000000000000000000000

00000000000000000000000000016492106522848313567845472606000000000000000000000000000

00000000000000000000000000017790104800073100000000000000000000000000000

0000000000000000000000000003060633745561809425571387911843122794475910967000000000000000000000000000

000000000000000000000000000000000000317324181474200278000000000024411551310171260326331433322497551636038285

1267595461317428405879449322182082889323612387513781222719017327021837211902169155995787240709411427234000000000000000000000000000

00000000000000000093519177625616045138224114739366942323191425116341532308000000000000000000000000000

00000000000000000093519177625616045138224114739366942323191425116341532308000000000000000000000000000

00000000000000000093519177625616045138224114739366942323191425116341532308000000000000000000000000000

2280000022800000000000000000000000000000000000000000000000000000000

2280000022800000000000000000000000000000000000000000000000000000000

2280000022800000000000000000000000000000000000000000000000000000000

10546096111577183671422629713288410582185001513682500718513050915458255311605862573069735820319731203152632671665121253652023996135348673950395104222181457604631261472656017493978786690100380486681147917277583344586262071140148632237396381011017288622675448463727374728176718964426035893656277509

12700000127002685240243714942322957472573108048860000000000000000007158431134480510625564301306000000000000000000

0000000000000000000000000000000000004352150203000170000000000000000000

1270000012700268524024371494232295747257310804886000000000000000000000000000000000000000000000

0000000000000000000000000000000000006723216134460210625564284306000000000000000000

217101311351301634127047223864185000000000444036626830998510853844016425696776848171830164451369457793836510298972640414954154198124343572824507111537712443101116054672592178256425690527088917865623068165263261292

0000000000000000000000000000000000002317016673069330937551204350118834782000000000000000000

254242021986282176154511100000000044403662683099851085384401642569677684817183016445136945779383651067279731080210644339856941289000000000000000000

000000000000000000000000000000000000000000000115377124431011000000000

00000000000000000000000000000000000000000000000000000016054672592178256425690527088917865623068165263261292

19168891330203031306871118414074000000000000000000000000000000000000000000000000000000

245904333850334247599753388735353771795510220377133218840181077874870520000000003171201999046057345701437421151308365303516143640120094102109804760819641512685707561289782703912958

0000000000000000000000000000000000000000000000000000009318370801908124765940505428697236018427

00000000000000000000000000000000000056682165853618931886359000000000000000000

0000000000000000000000000000000000003114521998844399345651401320261276485302915784000000000166215285626502630250728134384531

000000000000000000884018107787487052000000000000000000640120094102000000000

24590433385033424759975338873535377179551022037713321000000000000000000000000000000000000000000000

7972582881136153001059822168781531407572594131835852126977791109532444387110523049628312671284352645994425021354508330408458016104131190353156704548767876403732408421643636190543222741974753732126484351951892137129562116865661097000000000

0000000000000000000000000000000000001397718188257171150725670705628365262830640000000000000000000

000000000000000000000000000000000000000000000800042638066000000000

157321807184014315513200000000035225654147000114820000000000240035910441158100936089471832846000000000000000000

6399364811135689910594221656264014073298598037805795445850387857174949269742266266713735264599442496534508330408458016104131190353156704548767876403731177146316754373858512491699181213712952185648566491000000000

000000000174191593061762317657293951575735000000000000000000000000000000000000000000000

000000000253164987199137121453282064351336800000000000000000065297772616351142142242410000000000000000000

0000000000000000000000000000000000006357290112681605589070019040000000001359171910917162308342769913543259

000000000000000000000000000000000000635729011268160558907001904000000000000000000

0000000000000000000000000000000000000000000000000000001359171910917162308342769913543259

87209061764030219500000000000000000012238147381946171859172382000000000000000000000000000

872090617640302195000000000000000000000000000000000000000000000000000000

00000000000000000000000000012238147381946171859172382000000000000000000000000000

000000000432326787636797342141314380000000000000000003750159010219707000000000000000000

0000000000000000000000000000000000003750159010219707000000000000000000

00000000043232678763679734214131438000000000000000000000000000000000000000000000

2781302710274883000000000000000000000000000000000000000000000000000000

2781302710274883000000000000000000000000000000000000000000000000000000

122982543061582490152228216071818967221512212304787531848895100177131023560761712651956945693513124497234587721511698217815312740139027611038171011402025323752025364277584623103496672093211270519530254019224

122982543061582490152228216071818967221512212304787531848895100177131023560761712651956945693513124497234587721511698217815312740139027611038171011402025323752025364277086623103189670089171191816749253618547

0000000000000000000000000004993013122323458620321787000000000000000000000000000

00000000017661141965204122123047875634888606125016921811018200000000012740139027611038171068010144630489360600196632366913604

6156513168767001031203946167722011000255152815902160001530000000000000000000401901900020965420460589293017022

336140124537104331000000000000000000000000000000000000000000000000000000

278341142910806770000000003966171285194383991254425127005249401701511695014600000000010321252461156533223522762643129808694286791668083314941

000000000000000000000000000000000000000000000000000000498003072040478727814677

000000000000000000000000000000000000000000000000000000498003072040478727814677

11175288326916949300963071812473320343322167615498316216962274482745964162496485716139412237116117245581263083425751422924987996219144816219561807762623112085582239010924144488572351538271024011739217754221722148676417011259180770069239366291337988755552514511322562043261318581101182138180310

11175288326916949300963071812473320343322167615498316216962274482745964162496485716139412237116117245581263083425751422924987996219144816219561807762623112085582239010924144488572351538271024011739217754221722148676417011259180770069239366291337988755552514511322562043261318581101182138180310

11175288326916949300963071812473320343322167615498316216962274482745964162496485716139412237116117245581263083425751422924987996219144816219561807762623112085582239010924144488572351538271024011739217754221722148676417011259180770069239366291337988755552514511322562043261318581101182138180310

0000000008457617012551625261402682078000000000000000000000000000000000000000000000

0000000008457617012551625261402682078000000000000000000000000000000000000000000000

0000000008457617012551625261402682078000000000000000000000000000000000000000000000

221671193434440262135534959518471236444397423824816679287637888159170191347529057734916010156906657423416480104914950316884318783661223791427121154781627061544257198534071052744409264756457739673651048621568142102229128270135939515571251051121129765933218414257146642549612963355633923

221671193434440262135534959518471236444397423824816679287637888159170191347529057734916010156906657423416480104914950316884318783661223791427121154781627061544257198534071052744409264756457739673651048621568142102229128270135939515571251051121129765933218414257146642549612963355633923

00000000000000000000000000000000000046607810415481148398721904191237258059000000000000000000

0000000000000000000000000002378517742117956117033711532373000000000000000000000000000

48451175581686947013011208000000000000000000000000000000000000000000000000000000

0000000008924769058192417203921631000000000000000000000000000000000000000000000

00000000041490080898928757969813507011934752866571860134842462328419000000000000000000000000000000000000

000000000000000000000000000000000000443460215662403062000000000000000000

4322152026062839115366530000000008843804011062901481620000000000000000000000000001067034016665706153010451760

00000000000000000000000000000000000000000000054601004610000000000

212504190744435230265438058480461052742536000000000589815633659638221636610436488130028245854581120605142691975221615361540887198432541029013938763941456191556679492413753401901915774580305335145712559012000000000

000000000000000000000000000000000000000000000000000000102306559321837592139582534312963251132163

000000000000000000102111972341145000000000000000000000000000000000000

6551869395815779771191115001689522616575759859541426860290656368041751491966382872412265965385215992458877410110853922555378593766587106707703964858030868446459905098423652453145715025714950911992323361116625722479372272712470122016493858230600620097819313744414688435782080402113244123861272949429893356206678123594192672139650503519469507991714450

0000000000000000000000000000000000000000000000000000002630096701287122482204

0000000000000000000000000000000000000000000000000000002630096701287122482204

0000000000000000000000000000000000000000000000000000002630096701287122482204

150211520110752871741034623272000000000163681351476317558176479521723166393142996420727769414928323761600698450491226257546812221793403460003132712000000000

00000000000000000000000000038105299642072776328321000000000000000000000000000

0000000000000000000000000000000000009855937141808199644910120973326000000000000000000

000000000000000000897328383072021085215000000000615147490841857919218277000000000000000000

000000000000000000173303321725378372402000000000000000000000000000000000000

000000000000000000177000126160035000000000000000000460003132712000000000

150211520110752871741034623272000000000000000000120900068814600375000000000000000000000000000

0000000000000000001356110313659069081293220152514000000000000000000000000000000000000

25651160100110004643231608234684771526892644342272713843020353771511114813180954293159735439405543826244425646205458611959085272568472488813502363832392641592125119149562241262060113676316497604116085415599816671381253898320516512110775968220475362269215201208147083471154294198590235729350045363310213535116197307962354161223399

177920003177400000000000000000000000000000000000000000000000000000000

177920003177400000000000000000000000000000000000000000000000000000000

0000000000000000000000000000000000000000000000000000001798980563263890279650

0000000000000000000000000000000000000000000000000000001798980563263890279650

25473260098110004643231608234652997526892644342272713843020353771511114813180954293159735439405543826244425646205458611959085272568472488813502363832392641592125119149562241262060113676316497604116085415599816671381253898320516512110775968220475362269215201208147083471154294198410435719550045363254213209115808307962353882222749

00000000000000000000000000000000000063760142358116431822892123000000000000000000

00000000028071010782721126461274000000000000000000414180640003311000000000000000000

25473260098110004643231608234652997526892644341992013842920353771411107012908943033155134165405543826244425646205458611959085272568472488813502363832392641592125119149562241262060113676316496873716083515598316662680374881320198312048673637220475362269215201208147083471154294198410435719550045363254213209115808307962353882222749

000000000000000000000000000000000000514110298601207000000000000000000

4730010047200000000000000000000000000000000000000000000000000000000

4730010047200000000000000000000000000000000000000000000000000000000

4730010047200000000000000000000000000000000000000000000000000000000

0000000000000000000000000000000000000000000000000000002137163421532059337563489668365

0000000000000000000000000000000000000000000000000000002137163421532059337563489668365

0000000000000000000000000000000000000000000000000000002137163421532059337563489668365

2674112002265801000000000000000000000000000000000000000000000470011529972185409348391478

2674112002265801000000000000000000000000000000000000000000000470011529972185409348391478

000000000000000000000000000000000000000000000000000000470011529972185409348391478

1090000010801000000000000000000000000000000000000000000000000000000

2565112002255000000000000000000000000000000000000000000000000000000000

403361334125220749932959122521159528907718492026798012595712657113895134676927987246484313424104618301228017220000000000677229011001435121415124087649926571156138817862319123241402902412818982243799046317118160581465393853

4033613341252207499220291222241595287715183492267218125956114541080180000000000000000000000000005916143431017879891385392412317821039651945248818155558384358171

000000000922029122224159528771518349226721812595611454108018000000000000000000000000000000000000000000000

114360400074000000000000000000000000000000000000059161434310000000000

2890133012522009000000000000000000000000000000000000000000000000000000

00000000000000000000000000000000000000000000000000000017879891385392412317821039651945248818155558384358171

0000000001093029701362142876211203587700000000000000000000000000000000000012286612574116034147110659502214035682

00000000000000000000000000000000000000000000000000000012286612574116034147110659502214035682

00000000010930297013621428762112035877000000000000000000000000000000000000000000000

000000000000000000134676927987246484313424104618301228017220000000000000000000213561239870498265311558383877623000000000

000000000000000000134676927987246484313424104618301228017220000000000000000000213561239870498265311558383877623000000000

000000000000000000000000000000000000677229011001435100000000014694941902409410

000000000000000000000000000000000000677229011001435100000000014694941902409410

3757593366046332957374476143710139461953456227711742970549989215706081521095981059032187907079522171176418723399097330619314858168228722077294313336520964264824928669187857736612978970117715291121006227719611367090628210611532001313930367698637482851404201415366055225937114286736

0000000000000000000000000000000000007597423409601128212233686697284384497000000000000000000

0000000000000000000000000000000000007597423409601128212233686697284384497000000000000000000

0000000000000000000000000000000000004309113713657264477719483924720777221361277847000000000000000000

0000000000000000000000000000000000004309113713657264477719483924720777221361277847000000000000000000

775003114271039348000000000000000000420572477350768115871361021000000000000000000000000000

775003114271039348000000000000000000420572477350768115871361021000000000000000000000000000

13997201192631138701218000000000000000000000000000115859245918798147043310320316199257911926300000000046796169739711981062583189537

0000000000000000000000000000000000001133602429085001436828933107157782560118823000000000000000000

1399720119263113870121800000000000000000000000000024993012983364179642119044000000000046796169739711981062583189537

412210121371200289418142116393278121337130000000000302855021050322764143288349134174648000000000000000000000000000

0000000001814211639327812133713000000000090230234165113824864562006000000000000000000000000000

0000000000000000000000000002126250210480223534921501493612642000000000000000000000000000

4122101213712002894000000000000000000000000000000000000000000000000000000

3605753361946322953974199143288761945555767530321806649661405686080520758851059032187907079522171176418723399097330615865856942122921814645202932415980219519259464436377521318343821493551206390628210611532001313930363019631322754400231295564993220107095386199

3605753361946322953974199143288761945555767530321806649661405686080520758851059032187907079522171176418723399097330615865856942122921814645202932415980219519259464436377521318343821493551206390628210611532001313930363019631322754400231295564993220107095386199

43459124943180200000000000000000015041461746192227911246852129514068162120655076145143661979592106199661950000000004308557613909301174565216415

00000000000000000000000000015041461746192227911246852129514068162120655076145143000000000000000000000000000

00000000000000000000000000015041461746192227911246852129514068162120655076145143000000000000000000000000000

32795111732630100000000000000000000000000000000000000000000024682553223871853631998272

00000000000000000000000000000000000000000000000000000024682553223871853631998272

327951117326301000000000000000000000000000000000000000000000000000000

10664013210550100000000000000000000000000066197959210619966195000000000184030229152212638246118143

10664013210550100000000000000000000000000066197959210619966195000000000184030229152212638246118143

7380000173700000000000000000000000000000625901040005120000000000103513735265066307714476

3220000032200000000000000000000000000000625901040005120000000000103513735265066307714476

3220000032200000000000000000000000000000000000000000000000000000000

3220000032200000000000000000000000000000000000000000000000000000000

000000000000000000000000000000000000625901040005120000000000103513735265066307714476

000000000000000000000000000000000000625901040005120000000000000000000

000000000000000000000000000000000000000000000000000000103513735265066307714476

4160000141500000000000000000000000000000000000000000000000000000000

4160000141500000000000000000000000000000000000000000000000000000000

4160000141500000000000000000000000000000000000000000000000000000000

1814002000012100000000000000000000000000000000000000000000003173468053500021700

0000000000000000000000000000000000000000000000000000003173468053500021700

0000000000000000000000000000000000000000000000000000003173468053500021700

0000000000000000000000000000000000000000000000000000003173468053500021700

181400200001210000000000000000000000000000000000000000000000000000000

181400200001210000000000000000000000000000000000000000000000000000000

181400200001210000000000000000000000000000000000000000000000000000000

181400200001210000000000000000000000000000000000000000000000000000000

000000000149197565797145542636185807311521278720833395395231021533892412622843601760313116925735118546205758732559241846127121470122111101111129674842328351205843447831956639241

00000000000000000011521278720833395395231021530000000002573511854620575873255924184612712147012211110111000000000

00000000000000000011521278720833395395231021530000000002573511854620575873255924184612712147012211110111000000000

00000000000000000011521278720833395395231021530000000002573511854620575873255924184612712147012211110111000000000

00000000000000000000000000000000000022077110942210211175390000000000000000000

000000000000000000000000000000000000000000000147012211110111000000000

0000000000000000001120227872053272534411452121000000000235281115451948545124572367112322000000000000000000

0000000000000000003190036751116532000000000000000000000000000000000000

0000000001491975657971455426361858073000000000389241262284360176031311690000000000000000001129674842328351205843447831956639241

0000000001491975657971455426361858073000000000389241262284360176031311690000000000000000001129674842328351205843447831956639241

0000000001491975657971455426361858073000000000389241262284360176031311690000000000000000001129674842328351205843447831956639241

0000000001491975657971455426361858073000000000389241262284360176031311690000000000000000001129674842328351205843447831956639241

3506642837808634729275719105513818489169130646857671032216927529695810266076578458007396119361207150718321955350178813101987000000000572236289104592891037811462616874015191

3506642837808634729275719105513818489169130646857671032216927529695810266076578458007396119361207150718321955350178813101987000000000572236289104592891037811462616874015191

3506642837808634729275719105513818489169130646857671032216927529695810266076578458007396119361207150718321955350178813101987000000000572236289104592891037811462616874015191

0000000000000000000000000000000000008581403391005040000000000890537013663024548177

0000000000000000000000000000000000008581403391005040000000000890537013663024548177

3187837634727931574255119105513818489169130646857671032216927529695810266076578458007396818092111501043144925013176121438000000000381264494822437083578869489121344189

3159337634727931289255119105513818489169130646857671032216927529695810266076578458007396818092111501043144925013176121438000000000381264494822437083578869489121344189

1380000013800000000000000000000000000000000000000000000000000000000

1470000014700000000000000000000000000000000000000000000000000000000

00000000000000000000000000000000000000000000000000000014831671813540640815868123

00000000000000000000000000000000000000000000000000000014831671813540640815868123

6880000068800000000000000000000000000000000000000000000000000000000

1190000011900000000000000000000000000000000000000000000000000000000

5690000056900000000000000000000000000000000000000000000000000000000

2500523872467260000000000000000000000000002898272357450505100471194549000000000870912582041121161521831074382872

00000000000000000000000000000000000078256811191441816766131000000000000000000

00000000000000000000000000000000000010289913017617229148761980000000003032494100455550679304139311

1871001118400000000000000000000000000000000000000000000000000000000

124420312123024000000000000000000000000000000000000000000000000000000

00000000000000000000000000000000000010881171461551895315652220000000000000000000

106922064105302000000000000000000000000000000000000000000000000000000

000000000000000000000000000000000000000000000000000000567776410466610651504770243561
