# Supplementary material for: Relative abundance of ‘Candidatus Tenderia electrophaga’ is linked to cathodic current in an aerobic biocathode community
Source: Microb Biotechnol. 2017 Jul 11;11(1):98–111. doi: 10.1111/1751-7915.12757 (PMC5743799; doi:10.1111/1751-7915.12757)
Supplement: Supplementary file 7 [file MBT2-11-98-s007.html]

Javascript must be enabled to view this page.

richness


V12\_AllSamples
V12\_1031813
V12\_2021213
V12\_2031813
V12\_2040813
V12\_3040813
V12\_4021213
V12\_4032113
V12\_4040813
V3\_AllSamples
V3\_1031813
V3\_2021213
V3\_2031813
V3\_2040813
V3\_3040813
V3\_4021213
V3\_4032113
V3\_4040813
V4\_AllSamples
V4\_1031813
V4\_2021213
V4\_2031813
V4\_2040813
V4\_3040813
V4\_4021213
V4\_4032113
V4\_4040813
V5\_AllSamples
V5\_1031813
V5\_2021213
V5\_2031813
V5\_2040813
V5\_3040813
V5\_4021213
V5\_4032113
V5\_4040813
V6\_AllSamples
V6\_1031813
V6\_2021213
V6\_2031813
V6\_2040813
V6\_3040813
V6\_4021213
V6\_4032113
V6\_4040813
V78\_AllSamples
V78\_1031813
V78\_2021213
V78\_2031813
V78\_2040813
V78\_3040813
V78\_4021213
V78\_4032113
V78\_4040813
V9\_AllSamples
V9\_1031813
V9\_2021213
V9\_2031813
V9\_2040813
V9\_3040813
V9\_4021213
V9\_4032113
V9\_4040813

1202304138809228081495822202743024837164711812917857225484515203555398036032936742644810133995610104435726088699512384568008822501740821239311284319459591853585171610363358261028003048139949916007814084340455856933699643980533887025498305466349203554095938269247105948938715339852252463614787136941289114275134549601811733621120931125649110993458406657444831317996

5050000050500000000000000000000000000000000000000000000000000000000

5050000050500000000000000000000000000000000000000000000000000000000

640771518296336473341033350212107452000000000000000000014931162182103764426581183000000000000000000

640771518296336473341033350212107452000000000000000000014931162182103764426581183000000000000000000

640771518296336473341033350212107452000000000000000000014931162182103764426581183000000000000000000

640771518296336473341033350212107452000000000000000000014931162182103764426581183000000000000000000

256920322125373115661565001000000000000000000000014931162182103764426581183000000000000000000

256920322125373115661565001000000000000000000000014931162182103764426581183000000000000000000

29915121682952163184431785212974520000000000000000000000000000000000000000000000

29915121682952163184431785212974520000000000000000000000000000000000000000000000

8470000084700000000000000000000000000000000000000000000000000000000

5000000050000000000000000000000000000000000000000000000000000000000

3470000034700000000000000000000000000000000000000000000000000000000

000000000140014000000000000000000000000000000000000000000000000000000

000000000140014000000000000000000000000000000000000000000000000000000

000000000140014000000000000000000000000000000000000000000000000000000

000000000140014000000000000000000000000000000000000000000000000000000

4137139719646345263010594085688320441876851047456973563265141426143802200971748811282801371777291195760011446981201211566108500000000018352434518726811786125511950282951

4137139719646345263010594085688320441876851047456973563265141426143802200971748811282801371777291195760011446981201211566108500000000018352434518726811786125511950282951

4137139719646345263010594085688320441876851047456973563265141426143802200971748811282801371777291195760011446981201211566108500000000018352434518726811786125511950282951

4137139719646345263010594085688320441876851047456973563265141426143802200971748811282801371777291195760011446981201211566108500000000018352434518726811786125511950282951

3744137512613258150010263105688320441876851047456973500000000038022009717488112828013717753021846331915287308814034020000000001609542851822510129368211349002130

1156189137363227801488942198313021053007711703917810024911244817755383435846036578744355833974210022134774787533512380967905820341707071185061284189427789679583715210332948240977987988124329883117805164316655653833235093946553858315445875377328896253876137788945509248791715339842250461514676136941288113173249129490631718981100610122374310521508343477224881270613

500084433657001717551083311331523175411599214610642641911191703018331116914915631711314646369187823286561227011398326497847705191921711238814109927503083873043142554335534978614857402854252324332292716603121467091585602974713360835038023275517639926195543772694061705941432130816182276834033484692058442907518395368393305116188823517802

5974454978619920850212954413328867815426020620440025137101561313248892143771754623211138117181555297526517755361515614077025256369971608792863738638128255532023439386398257574952850779314556101131800595883139054601004610144964106142198284031425713744299513053415263

5974454978619920850212954413328867815426020620440025137101561313248892143771754623211138117181555297526517755361515614077025256369971608792863738638128255532023439386398257574952850779314556101131800595883139054601004610144964106142198284031425713744299513053415263

0000000000000000000000000001478935054200010000000000000000000000000000

128986163212418924976090623812040331822461121967365225120242393512230638477812294240641411096032350127324849103337847662593081712628985065271641580000000000000000000

15478067000905765502243836743197471312850000000000000000007108634511128412828606362000000000000000000

00000000000000000000000000000000000000000000000000000010025358236226193327575482653

00000000000000000067928122982324753126000000000000000000000000000000000000

00000000000000000000000000000000000025026282861006728309672347348530000000001353713271700064051927

0000000000000000000000000000000000005624208328841113241401574153200000000012986328017362322459708733130

1606551868397131037117000000000000000000000000000000000000000000000000000000

00000000000000000000000000016492106522848313567845472606000000000000000000000000000

00000000000000000000000000017790104800073100000000000000000000000000000

00000000000000000000000000015461120648710539198661815442429000000000000000000000000000

0000000000000000000000000000000000003173241814742002780000000000000000000

1267595461317428405879449322182082889323612387513781222719017327021837211902169155995787240709411427234000000000000000000000000000

00000000000000000093519177625616045138224114739366942323191425116341532308000000000000000000000000000

00000000000000000093519177625616045138224114739366942323191425116341532308000000000000000000000000000

00000000000000000093519177625616045138224114739366942323191425116341532308000000000000000000000000000

2280000022800000000000000000000000000000000000000000000000000000000

2280000022800000000000000000000000000000000000000000000000000000000

2280000022800000000000000000000000000000000000000000000000000000000

10546096111577183671422629713288410582185001339492484815452874712282198021566361002496235820319731203152632671665121253652023996135348673950395104222181457604631261472652360571950830712617802095464113531629554318222071140148632237396381011030212124716543664938593498047927790

12700000127000000000000000000000000000007158431134480510625564301306000000000000000000

0000000000000000000000000000000000004352150203000170000000000000000000

1270000012700000000000000000000000000000000000000000000000000000000

0000000000000000000000000000000000006723216134460210625564284306000000000000000000

21710131135130163412704722386418526852402437149423229574725731080488644403662683099851085384401642569677684817183016445136945779383651029897264041495415419812434357282450711153771244310111359171910917162308342769913543259

2542420219862821761545111000000000709126418541200000000067279731080210644339856941289000000000000000000

409480923910605866000000000000000000000000000000000000000000000000000000

2459043338503342475997533887353537717955102203771332188401810778748705200000000056682165853618931886359640120094102166215285626502630250728134384531

000000000000000000000000000000000000000000000000000000166215285626502630250728134384531

00000000000000000000000000000000000056682165853618931886359000000000000000000

000000000000000000884018107787487052000000000000000000640120094102000000000

24590433385033424759975338873535377179551022037713321000000000000000000000000000000000000000000000

79725828811361530010598221687815314075551751302427919507460353662849423053173049628312671284352645994425021354508330408458016104131190353156704548767876403731866021614524611502871371844332751025723241751892137129562116865661097000000000

000000000000000000000000000000000000108837795024340762712963436927186204122361000000000000000000

000000000000000000000000000000000000000000000800042638066000000000

157321807184014315513200000000035225654147000114820000000000697623931553159105000000000000000000

6399364811135689910594221656264014073298598037805795445850387857174949269742266266713735264599442496534508330408458016104131190353156704548767876403731177146316754373858512491699181213712952185648566491000000000

000000000253164987199137121453282064351336800000000000000000065297772616351142142242410000000000000000000

000000000000000000000000000000000000635729011268160558907001904000000000000000000

000000000000000000000000000000000000635729011268160558907001904000000000000000000

87209061764030219500000000000000000012238147381946171859172382000000000000000000000000000

872090617640302195000000000000000000000000000000000000000000000000000000

00000000000000000000000000012238147381946171859172382000000000000000000000000000

000000000432326787636797342141314380000000000000000003750159010219707000000000000000000

0000000000000000000000000000000000003750159010219707000000000000000000

00000000043232678763679734214131438000000000000000000000000000000000000000000000

2781302710274883000000000000000000000000000000000000000000000000000000

2781302710274883000000000000000000000000000000000000000000000000000000

122982543061582490152228216071818967221512212304787531848895100177131023560761712651956945693513124497234587721511698217815324580531034115512704658670175345111402025323752025364277584623103496672093211270519530254019224

122982543061582490152228216071818967221512212304787531848895100177131023560761712651956945693513124497234587721511698217815324580531034115512704658670175345111402025323752025364277086623103189670089171191816749253618547

0000000000000000000000000004993013122323458620321787000000000000000000000000000

000000000000000000606125016921811018200000000012740139027611038171068010144630489360600196632366913604

6156513168767001031203946167722011000255152815902160001530000000000000000000401901900020965420460589293017022

336140124537104331000000000000000000000000000000000000000000000000000000

27834114291080677000000000000000000000000000000000000000000000000000000

000000000000000000000000000000000000000000000000000000498003072040478727814677

000000000000000000000000000000000000000000000000000000498003072040478727814677

11175288326916949300963071812473320343322167615498316216962274482745964162496485716139412237116117245581263083425751422924987996219144816219561807762623112085582239010924144488572351538271024011739217754221722148676417011259180770069239366291337988755552514511322562043261318581101182138180310

11175288326916949300963071812473320343322167615498316216962274482745964162496485716139412237116117245581263083425751422924987996219144816219561807762623112085582239010924144488572351538271024011739217754221722148676417011259180770069239366291337988755552514511322562043261318581101182138180310

11175288326916949300963071812473320343322167615498316216962274482745964162496485716139412237116117245581263083425751422924987996219144816219561807762623112085582239010924144488572351538271024011739217754221722148676417011259180770069239366291337988755552514511322562043261318581101182138180310

0000000008457617012551625261402682078000000000000000000000000000000000000000000000

0000000008457617012551625261402682078000000000000000000000000000000000000000000000

0000000008457617012551625261402682078000000000000000000000000000000000000000000000

221671193434440262135534959518471236444397423824816679287637888159170191347529057734916010156906657423416480104914950316884318783661223791427121154781627061544257198534071052744409264756457739673651048621568142102229128270130533514571255901217184432651508474827116228555320415224364468819295215

221671193434440262135534959518471236444397423824816679287637888159170191347529057734916010156906657423416480104914950316884318783661223791427121154781627061544257198534071052744409264756457739673651048621568142102229128270130533514571255901217184432651508474827116228555320415224364468819295215

00000000000000000000000000000000000046607810415481148398721904191237258059000000000000000000

0000000000000000000000000002378517742117956117033711532373000000000000000000000000000

48451175581686947013011208000000000000000000000000000000000000000000000000000000

0000000008924769058192417203921631000000000000000000000000000000000000000000000

000000000000000000134842462328419000000000000000000000000000000000000

000000000000000000000000000000000000443460215662403062000000000000000000

4322152026062839115366530000000008843804011062901481620000000000000000000000000001067034016665706153010451760

2125041907444352302654380584804610527425364149008089892875796981350701193475286657186058981563365963822163661043648813002824585458112060514269197522161536154088719843254102901393876394145619155667949241375340190191577458030533514571255901216054672592178256425690527088917865623068165263261292

000000000000000000000000000000000000000000000000000000102306559321837592139582534312963251132163

000000000000000000102111972341145000000000000000000000000000000000000

6551869395815779771191115001689522616575759859541426860290656368041751491966382872412265965385215992458877410110853922555378593766587106707703964858030868446459905098423652453145715025714950911992323361116625722479372272712470122016493858230600620097819313744414688435782080402113244123861272949454304861337679835654518705282683727529224524351752735

0000000000000000000000000000000000000000000000000000002630096701287122482204

0000000000000000000000000000000000000000000000000000002630096701287122482204

0000000000000000000000000000000000000000000000000000002630096701287122482204

1502115201107528717410346232721093029701362142876211203587716368135147631755817647952172316649134304652695123442153541191295962449162150566439209181289126115341546632130271408927102524411551310171260326331433322497551636038285

00000000000000000000000000038105299642072776328321000000000000000000000000000

0000000000000000000000000000000000009855937141808199644910120973326000000000000000000

000000000000000000897328383072021085215000000000615147490841857919218277000000000000000000

000000000000000000173303321725378372402000000000000000000000000000000000000

000000000000000000177000126160035000000000000000000460003132712000000000

15021152011075287174103462327210930297013621428762112035877135611031365906908129322015251498205016223527481386235925834644363775213183438214935512063275302713776092324411551310171260326331433322497551636038285

000000000000000000000000000120900068814600375000000000000000000000000000

25651160100110004643231608234684771526892644342272713843020353771511114813180954293159735439405543826244425646205458611959085272568472488813502363832392641592125119149562241262060113676316497604116085415599816671381253898320516512110775968220475362269215201208147083471154294198590235729350045363310213535116197307962354161223399

177920003177400000000000000000000000000000000000000000000000000000000

177920003177400000000000000000000000000000000000000000000000000000000

0000000000000000000000000000000000000000000000000000001798980563263890279650

0000000000000000000000000000000000000000000000000000001798980563263890279650

25473260098110004643231608234652997526892644342272713843020353771511114813180954293159735439405543826244425646205458611959085272568472488813502363832392641592125119149562241262060113676316497604116085415599816671381253898320516512110775968220475362269215201208147083471154294198410435719550045363254213209115808307962353882222749

00000000000000000000000000000000000063760142358116431822892123000000000000000000

00000000028071010782721126461274000000000000000000414180640003311000000000000000000

25473260098110004643231608234652997526892644341992013842920353771411107012908943033155134165405543826244425646205458611959085272568472488813502363832392641592125119149562241262060113676316496873716083515598316662680374881320198312048673637220475362269215201208147083471154294198410435719550045363254213209115808307962353882222749

000000000000000000000000000000000000514110298601207000000000000000000

4730010047200000000000000000000000000000000000000000000000000000000

4730010047200000000000000000000000000000000000000000000000000000000

4730010047200000000000000000000000000000000000000000000000000000000

334149268714592268457366313928271103645246192202912222415952877151834922672181259561145410801813467692798724648431342410461830122801722015041461746192227911246852129514068162120655076145143431588373655726447782948392472077723140477784821415124087649926571156138817862319123241402902412818982243799046317118160581465393853

40336133412522074900000000000000000000000000000000000059161434310000000000

114360400074000000000000000000000000000000000000059161434310000000000

2890133012522009000000000000000000000000000000000000000000000000000000

00000000000000000000000000000000000000000000000000000012286612574116034147110659502214035682

00000000000000000000000000000000000000000000000000000012286612574116034147110659502214035682

0000000000000000003845947364850933516000000000000000000000000000000000000

0000000000000000003845947364850933516000000000000000000000000000000000000

000000000000000000000000000000000000677229011001435100000000014694941902409410

000000000000000000000000000000000000677229011001435100000000014694941902409410

2674112002265801000000000000000000000000000000000000000000000470011529972185409348391478

2674112002265801000000000000000000000000000000000000000000000470011529972185409348391478

000000000000000000000000000000000000000000000000000000470011529972185409348391478

1090000010801000000000000000000000000000000000000000000000000000000

2565112002255000000000000000000000000000000000000000000000000000000000

0000000000000000000000000000000000000000000000000000002137163421532059337563489668365

0000000000000000000000000000000000000000000000000000002137163421532059337563489668365

0000000000000000000000000000000000000000000000000000002137163421532059337563489668365

4201368251742732938445013875924437757117429705499892157060815210959810590321879070795221711764187233990973306183328576672286618537668331979209622289223451918334800088582598645333571228965422923760631279103416124131307367698637482851404201415366055225937114286736

0000000000000000000000000000000000007597423409601128212233686697284384497000000000000000000

0000000000000000000000000000000000007597423409601128212233686697284384497000000000000000000

775003114271039348000000000000000000420572477350768115871361021000000000000000000000000000

775003114271039348000000000000000000420572477350768115871361021000000000000000000000000000

1399720119263113870121800000000000000000000000000024993012983364179642119044000000000046796169739711981062583189537

1399720119263113870121800000000000000000000000000024993012983364179642119044000000000046796169739711981062583189537

4122101213712002894181421163932781213371300000000002046511049741139514974911582065000000000000000000000000000

0000000001814211639327812133713000000000090230234165113824864562006000000000000000000000000000

00000000000000000000000000011442110474074411547259000000000000000000000000000

4122101213712002894000000000000000000000000000000000000000000000000000000

268296784173269866140285916533155303218066496614056860805207588510590321879070795221711764187233990973306158658569421229218146452029324159802195192591133602429085001436828933107157782560118823631279103416124131307363019631322754400231295564993220107095386199

268296784173269866140285916533155303218066496614056860805207588510590321879070795221711764187233990973306158658569421229218146452029324159802195192591133602429085001436828933107157782560118823631279103416124131307363019631322754400231295564993220107095386199

434591249431802000000000000000000000000000661979592106199661950000000004308557613909301174565216415

32795111732630100000000000000000000000000000000000000000000024682553223871853631998272

00000000000000000000000000000000000000000000000000000024682553223871853631998272

327951117326301000000000000000000000000000000000000000000000000000000

10664013210550100000000000000000000000000066197959210619966195000000000184030229152212638246118143

10664013210550100000000000000000000000000066197959210619966195000000000184030229152212638246118143

7380000173700000000000000000000000000000625901040005120000000000103513735265066307714476

3220000032200000000000000000000000000000625901040005120000000000103513735265066307714476

3220000032200000000000000000000000000000000000000000000000000000000

3220000032200000000000000000000000000000000000000000000000000000000

000000000000000000000000000000000000625901040005120000000000103513735265066307714476

000000000000000000000000000000000000625901040005120000000000000000000

000000000000000000000000000000000000000000000000000000103513735265066307714476

4160000141500000000000000000000000000000000000000000000000000000000

4160000141500000000000000000000000000000000000000000000000000000000

4160000141500000000000000000000000000000000000000000000000000000000

1814002000012100000000000000000000000000000000000000000000003173468053500021700

0000000000000000000000000000000000000000000000000000003173468053500021700

0000000000000000000000000000000000000000000000000000003173468053500021700

0000000000000000000000000000000000000000000000000000003173468053500021700

181400200001210000000000000000000000000000000000000000000000000000000

181400200001210000000000000000000000000000000000000000000000000000000

181400200001210000000000000000000000000000000000000000000000000000000

181400200001210000000000000000000000000000000000000000000000000000000

0000000001491975657971455426361858073115212787208333953952310215354667426241179323621294145825735118546205758732559241846127121470122111101111129674842328351205843447831956639241

00000000000000000011521278720833395395231021531574330127433602981289257351185462057587325592418461271214701221111011120478691142338243595278283736

00000000000000000011521278720833395395231021531574330127433602981289257351185462057587325592418461271214701221111011120478691142338243595278283736

00000000000000000011521278720833395395231021531574330127433602981289257351185462057587325592418461271214701221111011120478691142338243595278283736

000000000000000000000000000000000000000000000147012211110111000000000

0000000000000000001120227872053272534411452121000000000235281115451948545124572367112322000000000000000000

000000000149197565797145542636185807300000000038924126228436017603131169000000000000000000924894152316928167603088329173835505

000000000149197565797145542636185807300000000038924126228436017603131169000000000000000000924894152316928167603088329173835505

000000000149197565797145542636185807300000000038924126228436017603131169000000000000000000924894152316928167603088329173835505

000000000149197565797145542636185807300000000038924126228436017603131169000000000000000000924894152316928167603088329173835505

3506642837808634729275719105513818489169130646857671032216927529695810266076578458007396119361207150718321955350178813101987000000000572236289104592891037811462616874015191

3506642837808634729275719105513818489169130646857671032216927529695810266076578458007396119361207150718321955350178813101987000000000572236289104592891037811462616874015191

3506642837808634729275719105513818489169130646857671032216927529695810266076578458007396119361207150718321955350178813101987000000000572236289104592891037811462616874015191

0000000000000000000000000000000000008581403391005040000000000890537013663024548177

0000000000000000000000000000000000008581403391005040000000000890537013663024548177

3187837634727931574255119105513818489169130646857671032216927529695810266076578458007396818092111501043144925013176121438000000000381264494822437083578869489121344189

3159337634727931289255119105513818489169130646857671032216927529695810266076578458007396818092111501043144925013176121438000000000381264494822437083578869489121344189

1380000013800000000000000000000000000000000000000000000000000000000

1470000014700000000000000000000000000000000000000000000000000000000

00000000000000000000000000000000000000000000000000000014831671813540640815868123

00000000000000000000000000000000000000000000000000000014831671813540640815868123

6880000068800000000000000000000000000000000000000000000000000000000

1190000011900000000000000000000000000000000000000000000000000000000

5690000056900000000000000000000000000000000000000000000000000000000

2500523872467260000000000000000000000000002898272357450505100471194549000000000870912582041121161521831074382872

00000000000000000000000000000000000078256811191441816766131000000000000000000

00000000000000000000000000000000000010289913017617229148761980000000003032494100455550679304139311

1871001118400000000000000000000000000000000000000000000000000000000

124420312123024000000000000000000000000000000000000000000000000000000

00000000000000000000000000000000000010881171461551895315652220000000000000000000

106922064105302000000000000000000000000000000000000000000000000000000

000000000000000000000000000000000000000000000000000000567776410466610651504770243561
