# Supplementary material for: Relative abundance of ‘Candidatus Tenderia electrophaga’ is linked to cathodic current in an aerobic biocathode community
Source: Microb Biotechnol. 2017 Jul 11;11(1):98–111. doi: 10.1111/1751-7915.12757 (PMC5743799; doi:10.1111/1751-7915.12757)
Supplement: Supplementary file 8 — Data S1. Supplemental Materials and Methods. Table S1. Biocathode MCL strain IDs and associated metrics. Table S2. Biocathode MCL metagenomic assembly metrics for both individual and pooled bioelectrochemical system (BES) sample reads. Table S3. Metagenomic binning and quality checks. Table S4. Relative abundance for all 7 replicate bioelectrochemical systems (BES) for which metagenomic reads were generated using Metaphyler, Kraken, and blastn at the genus, family, and order levels. Table S5. Correlation analysis comparing Metaphyler, Kraken and blastn analysis to each other or to current for the top 25 orders identified across all samples (pooled) representing at least 0.5% of total abundance (“‐” indicates at least one method had no counts in the specified order level group). Table S6. Number of read pairs generated for each 16S rRNA gene hypervariable region for each bioelectrochemical systems (BES). Table S7. OTUs found for each 16S rRNA gene hypervariable region using either CD‐HIT or mothur. Table S8. Relative abundance predicted by 16S rRNA gene amplicon sequencing of each hypervariable region for eight replicate bioelectrochemical systems (BES) using OTUs generated by CD‐HIT with RDP classifier. Table S9. Relative abundance estimates of the 20 most dominant bin genomes based on metagenomic sequencing and 16S rRNA gene sequencing. Table S10. Primer sequences for 16S rRNA gene hypervariable regions used in this study. [file MBT2-11-98-s008.docx]

**Supplemental Materials and Methods**

**Electrochemical reactors**

Electrical connection to the working electrodes was made with titanium wire lead inserted into the chamber through septa in the screw top lids. Counter electrodes were graphite rods (radius 0.3 cm, height 6 cm; total geometric surface area of 0.00120 m^2^) inserted through side ports of the reactor and held in place by rubber gaskets. Partially assembled electrochemical reactors were autoclaved. Reference electrodes (Ag/AgCl, 3 M NaCl, BASinc.) were sterilized separately in 10% bleach, inserted through side ports of the sterilized reactor and held in place by rubber gaskets. The reactors were filled to minimize headspace with artificial seawater medium (ASW) ([Schubbe et al., 2009](#_ENREF_15)) containing 27.50 g NaCl, 3.80 gMgCl_2_•6H_2_O, 6.78 g MgSO_4_•H_2_O, 0.72 g KCl, 0.62 g NaHCO_3_, 2.79 g CaCl_2_•2H_2_O, 1.00 g NH_4_Cl, 0.05 g K_2_HPO_4_, and 1 ml Wolfe’s Trace Mineral Solution per liter. The medium was brought to a final pH of 6.1-6.5 with CO_2_. Background current during chronoamperometry and CV (0.300 V to -0.125 V vs. Ag/AgCl and back to 0.300 V at a scan rate of 0.0002 V/s) was recorded for each reactor before inoculation and any subsequent CV recorded used the same parameters. E_M_ (i_L_/2) = 0.219 V vs. Ag/AgCl.

**Draft assemblies of biocathode isolates.** Isolation of strains 3 (CP1) and 4 (CP2c) were previously reported ([Wang et al., 2015](#_ENREF_17)). Strains and 2 (CP4), 5 (CP2a), and 8 (CP2b) were enriched in an identical manner as 3 and 4 by resuspending electrode scrapings from the Biocathode MCL surface into 1 mL ASW and spreading 30-50 μL of suspension onto marine broth agar (Difco). Following incubation at 30°C, colonies of varying morphology were restreaked several times before selection for growth with either full or half-strength (diluted with ASW) marine broth (Difco). Strains 7 (ND6WE1B), 9 (ND6WE1A1), and 10 (ND6WE1A3) were isolated as above following an initial enrichment step in iron gradient tubes ([Wang et al., 2015](#_ENREF_17)). DNA from these eight heterotrophic isolates was collected (Wizard® Genomic DNA Purification Kit) and subjected to sequencing using the Miseq platform (Table S2). Approximately 1.0 ng of high-quality DNA was processed using an Illumina Nextera® XT sample preparation kit following manufacturer’s instructions (Illumina, San Diego, CA, USA). Final libraries were validated and sequenced at 2x100 base pairs (bp) (paired-end reads). The raw data was trimmed using Sickle v1.33 ([Joshi et al., 2011](#_ENREF_11)), error corrected using Ecc of the Bbmap utility([Bushnell, 2016](#_ENREF_5)) v34.8, and then assembled using Ray v2.3.1 ([Boisvert et al., 2012](#_ENREF_3)). The k-mer and node coverage parameters were varied to generate several candidate Ray assemblies. A k-mer of 61 and minimum coverage depth of 8 was found to be optimal, resulting in a low number of contigs with very low number of errors, and was used to produce 8 draft isolate genomes. Isolate strains 2 and 3 (classified as *Labrenzia* and *Marinobacter* respectively) were also sequenced using the PacBio sequencing platfor. For those isolates, there was good agreement between the closed PacBio assemblies and Ray-MetaBAT case 1b.

No isolates could be obtained for strains 1 and 6. Closed genomes for these strains were recovered using PacBio sequencing assemblies. We have previously reported the genome for strain 1 as “*Candidatus* Tenderia electrophaga” ([Eddie et al., 2016](#_ENREF_10)). The genome of strain 6 is reported for the first time here. For metagenomic sequencing using the PacBio platform, total Biocathode MCL genomic DNA from a source reactor was extracted using the Qiagen Puregene Yeast/Bacteria Kit (Qiagen) following the manufacturer’s suggested protocol for Gram negative bacteria. Quality of the purified DNA was assessed on an agarose gel prior to library preparation. SMRTbell libraries with 5-10kb inserts were prepared following low-input protocols ([Protocol, 2014](#_ENREF_14)) and sequenced on 9 SMRT cells using P6-C4 chemistry on a PacBio RS II. Contigs were assembled using the Hierarchical Genome Assembly Process ([Chin et al., 2013](#_ENREF_8)) resulting in a 4.7 Mb circular chromosome, together with many small contigs from lower abundance community members. Base composition of the chromosome was 67.1 mol% G+C.

**Metagenome assembly quality checks.** AMPHORA2 identifies and assigns taxonomy using 31 different housekeeping genes ([Wu and Scott, 2012](#_ENREF_18)) (Table S3). A consensus taxonomic assignment was selected for each Biocathode MCL bin genome based on the closest match represented by the majority of these housekeeping genes at the lowest taxonomic level for which there was agreement. Putative bin genomes were quality assessed using both CheckM v1.05 ([Parks et al., 2015](#_ENREF_13)) and MUMmer v3.23([Delcher et al., 1999](#_ENREF_9)). CheckM assigns a phylogenetic identity to a putative bin genome and then uses the nearest neighbors in its database to estimate completeness. MUMmer was used to align the contigs of individual meta-bin genomes to each of the isolate strain sequences. A contig had to have a section of sequence that was >1000 bps long with 99% identity to be counted as matching a strain sequence. Bin genomes with ≥4000 bps and 50% identity were considered to be related to isolate strains.

**16S rRNA gene sequence long read database**. The ssu_finder tool as part of the CheckM software package was run on all predicted bin genomes to find possible 16S rRNA sequences. A total of 75 16S rRNA gene sequences (>1000 bp) were extracted from all Ray assemblies and 32 from all the IDBA-UD assemblies (Table 2). These sequences were de-replicated by aligning (BLAST) to each other resulting in 22 unique long 16S rRNA gene sequences from all assembly cases. Seven of these 22 could be aligned (BLAST) (99% identity) to biocathode isolate genomes confirming that they were correctly assembled. At the same time, the 16S rRNA gene sequences (900-1000 bp lengths) from a previously generated 16S rRNA gene clone library of a Biocathode MCL progenitor bioelectrochemical system ([Strycharz-Glaven et al., 2013](#_ENREF_16)) were clustered at 99% similarity generating a separate database of 123 unique clones. The 22 16S sequences extracted from metagenomic bins were then aligned (BLAST) to this clone database. All aligned (97% identity) with clones, further validating 16S sequences extracted from metagenomic data. The final long 16S reference sequence database retained whichever of the two sequences, 16S clone or metagenomic sequences, was longer.

**Supplemental Figure legends and Tables**

**Table S1.** Biocathode MCL strain IDs and associated metrics.

**Table S2.** Biocathode MCL metagenomic assembly metrics for both individual and pooled bioelectrochemical system (BES) sample reads.

**Table S3 (Excel spreadsheet).** Metagenomic binning and quality checks. Bin genome ID numbers associated with each assembly and clustering method for each bioelectrochemical system (BES) based on both read assembly for individual reactors and pooled read assembly. Bin characteristics (i.e. GC content, genome size, # of contigs) are given for each bin genome as well as coverage depth, CheckM metrics and AMPHORA2 metrics. Also given is the number of contigs from each bin genome that can be matched with a Biocathode MCL isolate strain genome.

**Table S4 (Excel spreadsheet).** Relative abundance for all 7 replicate bioelectrochemical systems (BES) for which metagenomic reads were generated using Metaphyler, Kraken, and blastn at the genus, family, and order levels. Data shown in this table is identical to that depicted graphically in Figure S2.

**Table S5 (Excel spreadsheet).** Correlation analysis comparing Metaphyler, Kraken and blastn analysis to each other or to current for the top 25 orders identified across all samples (pooled) representing at least 0 .5% of total abundance (“-“ indicates at least one method had no counts in the specified order level group).

**Table S6.** Number of read pairs generated for each 16S rRNA gene hypervariable region for each bioelectrochemical systems (BES).

**Table S7.** OTUs found for each 16S rRNA gene hypervariable region using either CD-HIT or mothur. The number of OTUs representing taxa estimated to be at 0.0075% relative abundance or greater are shown for mothur. The number of OTUs representing 0.5% or greater relative abundance are shown for CD-HIT and for mothur.

**Table S8 (Excel spreadsheet).** Relative abundance predicted by 16S rRNA gene amplicon sequencing of each hypervariable region for eight replicate bioelectrochemical systems (BES) using OTUs generated by CD-HIT with RDP classifier. The OTU ID number is given in column 1 and RDP taxonomic classification of longer 16S sequence to which OTU maps is listed in column 2 and is color coded across all variable regions to indicate that the OTUs mapped to the same long sequence even when the taxonomy of the individual variable region differed using RDP. For comparison, metagenomic bin genomes are also included with AMPHORA2 classification to show how CD-HIT OTUs mapped for each reactor with available metagenomic sequencing.

**Table S9 (Excel spreadsheet).** Relative abundance estimates of the 20 most dominant bin genomes based on metagenomic sequencing and 16S rRNA gene sequencing. Relative abundance is given based on read coverage generated from metagenomic sequencing, relative abundance of each bin genome scaled by known or predicted 16S rRNA gene copy number, and average 16S OTU read counts that could be linked to longer 16S sequences mapping to bin genomes.

**Table S10.** Primer sequences for 16S rRNA gene hypervariable regions used in this study.

**Figure S1.** Computational workflow overview.

**Figure S2 (Krona plot, download file before viewing in browser).** Interactive Krona plots depicting relative abundance of 20 resolved dominant Ray-MetaBAT case 1b bin genomes to each other classified by AMPHORA2 (i.e. Sphingomonadaceae should be Kordiimonadaceae as noted in the main text). Unresolved bin genomes are expected to make up less than 1% of the Biocathode MCL community in each reactor since bin genomes shown here make up at least 1% relative abundance. Sample IDs are for each individual bioelectrochemical system (BES).

**Figure S3a-c (Krona plot, download file before viewing in browser).** Interactive Krona plots depicting relative abundance of predicted taxa at the genus, family, and order levels resolved using Metaphyler (a), Kraken (b), and blastn (c).

**Figure S4a-b (Krona plot, download file before viewing in browser).** Interactive Krona plots depicting relative abundance predicted by 16S rRNA gene amplicon sequencing of each hypervariable region for eight replicate bioelectrochemical systems (BES) using OTUs generated by CD-HIT with RDP classifier (a) or mothur (b).

**Table S1.** Biocathode MCL isolate strains and associated metrics.

| Strain ID number^1,2^ | Isolate or genome designation | Genome size (Mbp) | # of contigs | % Complete | Classification using housekeeping genes (AMPHORA2)^3^ | Bin genome ID | Number of contigs mapping to genome | 16S rRNA ID# (in-house database) | 16S rRNA gene RDP classification (in-house database or bin genome)^3^ | 16S variable regions with matching OTUs (Illumina amplicon sequencing) | Reference |
| --- | --- | --- | --- | --- | --- | --- | --- | --- | --- | --- | --- |
| 1 | "*Ca*. Tenderia electrophaga" | 3.83 | 2 | 99.63 | *Chromatiaceae* (f) | k61mbatsc01 | 72 | 2 | *Thiohalobacter* (g) | All | Eddie et al., 2016 IJSEM |
| 2 | *Labrenzia* sp. CP4 | 6.05 | 2 | 99.37 | *Polymorphum gilvum* (s) | k61mbatsc09 | 27 | 1 | *Labrenzia* (g) | All | Wang et al., 2016 Genome A |
| 3 | *Marinobacter* sp. CP1 | 4.77 | 1 | 100 | *Marinobacter hydrocarbonoclasticus* (s) | k61mbatsc03m, k61mbatsc10m | 187, 455 | 3 | *Marinobacter* (g) | All | Wang et al., 2015a Genome A |
| 4 | CP2c | 4.45 | 1401 | 98.28 | *Alcanivorax borkumensis* (s) | k61mbatsc07 | 16 | 12 | *Alcanivorax* (g) | All |  |
| 5 | CP2a | 4.27 | 173 | 100 | *Muricauda ruestringensis* (s) | k61mbatsc29m | 267 | 14 | *Muricauda* (g) | 1/2,3,5,6,9 |  |
| 6 | *Anderseniella* sp. | 4.7 | 1 | 99.94 | *Alphaproteobacteria* (c) | k61mbatsc26 | 398 | 8 | *Anderseniella* (g) | not in 7/8 |  |
| 7 | ND6WE1B | 3.83 | 234 | 98.16 | *Hyphomonas neptunium* (s) | k61mbatsc25, k61mbatsc11, k61mbatsc32 | 11, 14, 195 | no mapping | *16S too short* | - |  |
| 8 | CP2b | 4.12 | 96 | 99.34 | *Flavobacteriaceae* (f) | - | - | 18 | *Muriicola* (g) | 1/2,4,6,9 |  |
| 9 | ND6WE1A1 | 2.32 | 77 | 99.34 | *Rothia* (g) | - | - | no mapping | *16S too short* | - |  |
| 10 | ND6WE1A3 | 2.31 | 510 | 98.34 | *Micrococcaceae* (f) | - | - | no mapping | *Branchiibius* (g) | - |  |
| ^1^All strains except 1 and 6 have been cultivated in isolation from Biocathode MCL and sequenced using Illumina Miseq technology. | | | | | | | | | |  |  |
| ^2^Strains 1 and 6 recovered using PacBio sequencing assemblies and the metrics for that sequencing are reported. | | | | | | | | |  |  |  |
| ^3^g=genus, f=family, s=species, c=class | | |  |  |  |  |  |  |  |  |  |

**Table S2.** Biocathode MCL metagenomic assembly metrics for both individual and pooled bioelectrochemical system (BES) sample reads.

| **BES reactor ID** | **Assembly** | **Total BPs** | **contig > 0 bp** | | **contig > 1 Kbp** | | **# 16S in contigs** | |
| --- | --- | --- | --- | --- | --- | --- | --- | --- |
|  |  |  | **#** | **N50** | # | N50 | All | >1Kbp |
| **Pooled** | Ray | 99,888,322 | 32,079 | 29,903 | 21,624 | 43,468 | 42 | 19 |
|  | IDBA_UD | 202,987,673 | 97,479 | 11,701 | 27,309 | 21,945 | 162 | 3 |
| **1031813** | Ray | 31,611,341 | 10,663 | 20,091 | 4,816 | 29,427 | 25 | 10 |
|  | IDBA_UD | 62,014,181 | 24,196 | 36,002 | 6,939 | 67,318 | 51 | 5 |
| **2021213** | Ray | 32,647,483 | 6,977 | 31,134 | 2,872 | 34,653 | 19 | 6 |
|  | IDBA_UD | 61,616,180 | 20,816 | 29,166 | 6,383 | 45,602 | 39 | 3 |
| **2031813** | Ray | 25,728,325 | 9,070 | 24,233 | 3,240 | 55,617 | 23 | 7 |
|  | IDBA_UD | 80,574,766 | 32,917 | 17,683 | 10,767 | 34,920 | 51 | 4 |
| **3042313** | Ray | 19,372,712 | 9,330 | 4,257 | 4,000 | 7,936 | 9 | 5 |
|  | IDBA_UD | 57,372,524 | 25,018 | 16,050 | 6,796 | 30,761 | 40 | 5 |
| **4040813** | Ray | 31,209,425 | 10,128 | 9,656 | 5,517 | 12,974 | 24 | 9 |
|  | IDBA_UD | 75,860,536 | 38,250 | 8,549 | 11,384 | 21,632 | 49 | 4 |
| **4021213** | Ray | 36,782,652 | 7,267 | 21,395 | 4,186 | 23,640 | 22 | 8 |
|  | IDBA_UD | 55,875,802 | 16,542 | 40,179 | 5,173 | 54,193 | 31 | 6 |
| **4032113** | Ray | 39,546,228 | 10,730 | 20,107 | 5,944 | 26,278 | 21 | 11 |
|  | IDBA_UD | 87,589,653 | 44,573 | 11,571 | 12,197 | 40,765 | 82 | 2 |

**Table S6.** Number of read pairs generated for each 16S rRNA gene hypervariable region for each bioelectrochemical systems (BES).

| BES reactor ID | V1/2 | V3 | V4 | V5 | V6 | V7/8 | V9 |
| --- | --- | --- | --- | --- | --- | --- | --- |
| 1031813 | 516,131 | 1,028,265 | 731,647 | 1,734,621 | 750,537 | 243,482 | 1,223,222 |
| 2021213 | 222,598 | 237,459 | 498,518 | 1,657,359 | 747,879 | 208,665 | 266,890 |
| 2031813 | 814,326 | 826,466 | 669,695 | 1,485,381 | 1,051,051 | 199,120 | 1,471,643 |
| 2040813 | 818,260 | 633,796 | 831,559 | 1,263,350 | 1,061,144 | 164,835 | 1,617,285 |
| 3040813 | 1,090,427 | 879,111 | 737,086 | 1,537,749 | 182,022 | 484,609 | 1,442,458 |
| 4021213 | 405,308 | 670,565 | 808,268 | 1,299,629 | 943,010 | 367,349 | 1,043,064 |
| 4032113 | 863,353 | 298,303 | 662,560 | 208,486 | 741,376 | 132,796 | 1,088,842 |
| 4040813 | 943,541 | 764,275 | 603,110 | 936,411 | 948,710 | 140,086 | 1,778,697 |

**Table S7.** OTUs found for each 16S rRNA gene hypervariable region using either CD-HIT or mothur. The number of OTUs representing taxa estimated to be at 0.0075% relative abundance or greater are shown for mothur. The number of OTUs representing 0.5% or greater relative abundance are shown for CD-HIT.

| Variable  Region | CD-HIT  All | CD-HIT  0.5% | mothur  All | mothur  0.0075% | mothur  0.5% |
| --- | --- | --- | --- | --- | --- |
| V1/2 | 69 | 17 | 159 | 75 | 14 |
| V3 | 29 | 16 | 354 | 74 | 17 |
| V4 | 46 | 11 | 230 | 47 | 11 |
| V5 | 33 | 12 | 79 | 71 | 10 |
| V6 | 53 | 16 | 440 | 74 | 18 |
| V7/8 | 21 | 8 | 114 | 66 | 10 |
| V9 | 40 | 12 | 64 | 54 | 12 |

**Table S10.** Primer sequences for 16S rRNA gene hypervariable regions used in this study.

| **Names** | **Sequence (5’ → 3’)** | **Region(s)** | **Size (bp)** | **PCR conditions** | **Reference(s)** |
| --- | --- | --- | --- | --- | --- |
| F27 | ACGTCAGAGTTTGATCMTGGCTCAG | V1 & 2 | 326 | 95^o^C, 3’; 30 cycles of 95^o^C, 30”, 55^o^C, 45”, 72^o^C, 90”; 72^o^C, 7’. | ([Kunin et al., 2010](#_ENREF_12)) |
| R342 | CTGCTGCSYCCCGTAG |  |  |  |  |
| F357 | ACTCCTACGGGAGGCAGCAG | V3 | 162 | 95^o^C, 10’; 30 cycles of 95^o^C, 1’, 50^o^C, 1’, 72^o^C, 1’; 72^o^C, 7’ | ([Bartram et al., 2011](#_ENREF_2); [Cai et al., 2013](#_ENREF_6)) |
| R518 | ATTACCGCGGCTGCTGG |  |  |  |  |
| F515 | GTGCCAGCMGCCGCGGTAA | V4 | 292 | 94^o^C, 3’; 35 cycles of 94^o^C, 45”, 50^o^C, 60”, 72^o^C, 90”; 72^o^C, 10’ | ([Caporaso et al., 2011](#_ENREF_7)) |
| R806 | GGACTACHVGGGTWTCTAAT |  |  |  |  |
| F786 | GATTAGATACCCTGGTAG | V5 | 141 | 94^o^C, 2’; 30 cycles of 94^o^C, 1’, 50^o^C, 1’, 72^o^C, 1’; 72^o^C, 10’ | ([Baker et al., 2003](#_ENREF_1); [Bokulich et al., 2012](#_ENREF_4)) |
| R926 | CCGTCAATTCMTTTGAGTTT |  |  |  |  |
| F926 | AAACTCAAAKGAATTGACGG | V6 | 121 | 94^o^C, 5’; 35 cycles of 94^o^C, 50”, 40^o^C, 30”, 72^o^C, 90”; 72^o^C, 5’ | ([Baker et al., 2003](#_ENREF_1); [Cai et al., 2013](#_ENREF_6)) |
| R1046 | CGACAGCCATGCANCACCT |  |  |  |  |
| F1114 | GCAACGAGCGCAACCC | V7 & 8 | 379 | 94^o^C, 5’; 35 cycles of 94^o^C, 50”, 40^o^C, 30”, 72^o^C, 90”; 72^o^C, 5’ | ([Kunin et al., 2010](#_ENREF_12)) |
| R1492 | TACGGYTACCTTGTTACGACTT |  |  |  |  |
| F1406 | TGYACACACCGCCCGT | V9 | 105 | 94^o^C, 2’; 30 cycles of 94^o^C, 1’, 50^o^C, 60”1’, 72^o^C, 1’; 72^o^C, 10’ | ([Baker et al., 2003](#_ENREF_1)) |
| 1510R | GGTTACCTTGTTACGACTT |  |  |  |  |

**References:**

Baker, G.C., Smith, J.J., and Cowan, D.A. (2003) Review and re-analysis of domain-specific 16S primers. *Journal of Microbiological Methods* **55**: 541-555.

Bartram, A.K., Lynch, M.D.J., Stearns, J.C., Moreno-Hagelsieb, G., and Neufeld, J.D. (2011) Generation of Multimillion-Sequence 16S rRNA Gene Libraries from Complex Microbial Communities by Assembling Paired-End Illumina Reads (vol 77, pg 3846, 2011). *Appl Environ Microbiol* **77**: 5569-5569.

Boisvert, S., Raymond, F., Godzaridis, E., Laviolette, F., and Corbeil, J. (2012) Ray Meta: scalable de novo metagenome assembly and profiling. *Genome Biology* **13**.

Bokulich, N.A., Joseph, C.M.L., Allen, G., Benson, A.K., and Mills, D.A. (2012) Next-Generation Sequencing Reveals Significant Bacterial Diversity of Botrytized Wine. *Plos One* **7**.

Bushnell, B. (2016) BBMap short read aligner.

Cai, L., Ye, L., Tong, A.H.Y., Lok, S., and Zhang, T. (2013) Biased Diversity Metrics Revealed by Bacterial 16S Pyrotags Derived from Different Primer Sets. *Plos One* **8**.

Caporaso, J.G., Lauber, C.L., Walters, W.A., Berg-Lyons, D., Lozupone, C.A., Turnbaugh, P.J. et al. (2011) Global patterns of 16S rRNA diversity at a depth of millions of sequences per sample. *Proceedings of the National Academy of Sciences of the United States of America* **108**: 4516-4522.

Chin, C.S., Alexander, D.H., Marks, P., Klammer, A.A., Drake, J., Heiner, C. et al. (2013) Nonhybrid, finished microbial genome assemblies from long-read SMRT sequencing data. *Nat Methods* **10**: 563-+.

Delcher, A.L., Kasif, S., Fleischmann, R.D., Peterson, J., White, O., and Salzberg, S.L. (1999) Alignment of whole genomes. *Nucleic Acids Research* **27**: 2369-2376.

Eddie, B.E., Wang, Z., Malanoski, A.P., hall, R.J., Oh, S.D., Heiner, C. et al. (2016) Description of "Candidatus Tenderia electrophaga", an uncultivated electroautotroph from a biocathode enrichment. *Int J Syst Evol Microbiol*.

Joshi, K., Anjum, F., Gowda, S., Damania, D., Graham-Hill, S., Gillette, P. et al. (2011) Uric Acid as a Potential Biomarker of Pulmonary Arterial Hypertension in Patients with Sickle Cell Disease. *Indian Journal of Hematology and Blood Transfusion* **27**: 96-100.

Kunin, V., Engelbrektson, A., Ochman, H., and Hugenholtz, P. (2010) Wrinkles in the rare biosphere: pyrosequencing errors can lead to artificial inflation of diversity estimates. *Environmental Microbiology* **12**: 118-123.

Parks, D.H., Imelfort, M., Skennerton, C.T., Hugenholtz, P., and Tyson, G.W. (2015) CheckM: assessing the quality of microbial genomes recovered from isolates, single cells, and metagenomes. *Genome Res* **25**: 1043-1055.

Protocol, P.S.S. (2014) 10 kb to 20 kb template preparation and 316 sequencing with low-input DNA.

Schubbe, S., Williams, T.J., Xie, G., Kiss, H.E., Brettin, T.S., Martinez, D. et al. (2009) Complete Genome Sequence of the Chemolithoautotrophic Marine Magnetotactic Coccus Strain MC-1. *Appl Environ Microbiol* **75**: 4835-4852.

Strycharz-Glaven, S.M., Glaven, R.H., Wang, Z., Zhou, J., Vora, G.J., and Tender, L.M. (2013) Electrochemical Investigation of a Microbial Solar Cell Reveals a Nonphotosynthetic Biocathode Catalyst. *Appl Environ Microbiol* **79**: 3933-3942.

Wang, Z., Leary, D.H., Malanoski, A.P., Li, R.W., Hervey, W.J., Eddie, B.J. et al. (2015) A Previously Uncharacterized, Nonphotosynthetic Member of the Chromatiaceae Is the Primary CO2-Fixing Constituent in a Self-Regenerating Biocathode. *Appl Environ Microbiol* **81**: 699-712.

Wu, M., and Scott, A.J. (2012) Phylogenomic analysis of bacterial and archaeal sequences with AMPHORA2. *Bioinformatics* **28**: 1033-1034.
